# CLN7 protein functions at the interface between endolysosomes and stress granules to promote cell survival

**DOI:** 10.1038/s41419-025-08063-4

**Published:** 2025-10-31

**Authors:** Aseel Sharaireh, Marta Guevara-Ferrer, Anna M. Ludlaim, Jonathan D. Humphries, Alexander M. Phillips, Andrew W. Dowsey, Zehan Zhang, John R. Counsell, Richard D. Unwin, Sara E. Mole, Ahad A. Rahim, Tristan R. McKay

**Affiliations:** 1 Department of Life Sciences, Manchester Metropolitan University, Manchester, UK; 2 Restorative Dentistry Department, School of Dentistry, University of Jordan, Amman, Jordan; 3 Department of Electrical Engineering & Electronics, University of Liverpool, Liverpool, UK; 4 Department of Population Health Sciences, Faculty of Health and Life Sciences, University of Bristol, Bristol, UK; 5 Department of Targeted Intervention, Division of Surgery & Interventional Science, University College London, London, UK; 6 Division of Cancer Sciences, School of Medical Sciences, Faculty of Biology, Medicine and Health, The University of Manchester, Manchester, UK; 7 Great Ormond Street Institute of Child Health, University College London, London, WC1E 6BT UK; 8 Department of Pharmacology, UCL School of Pharmacy, University College London, London, UK

**Keywords:** Cellular neuroscience, Mechanisms of disease

## Abstract

Inherited biallelic mutations in the *CLN7* gene result in the variant late infantile onset neuronal ceroid lipofuscinosis, a subtype of Batten disease (BD), a severe and fatal childhood neurodegenerative disease. Intriguingly, CLN7 genetic variants have also been associated with retinopathies, amyotrophic lateral sclerosis, and frontotemporal dementia. *CLN7* encodes a transmembrane protein localizing to endolysosomal membranes with outward-facing chloride channel activity. Loss of CLN7 function results in cortical neurons accumulating swollen lipofuscin-containing lysosomes, leading to neuroinflammation and neurodegeneration. The molecular mechanisms underlying CLN7 BD neuropathology are not completely understood. We have generated iPSC lines from two CLN7 BD patients and age-matched unaffected controls to interrogate intracellular molecular phenotypes in iPSC-derived neural progenitor cells (iNPC). Taking a multi-omics approach we have identified disease-modified activities in endolysosomal transport in iNPC^BD^ that lead to lysosomal dysfunction and decreased mitophagy, resulting in the accumulation of metabolically defective mitochondria. We further observe a breakdown in nuclear functions that centre on RNA processing and nuclear export, linking to CLN7 protein interactions at the stress granule. We have identified dual and distinct functions for CLN7, promoting cell survival during the cellular stress response. CLN7 loss of function in BD results in neuronal apoptosis.

## Introduction

The Neuronal Ceroid Lipofuscinoses, also collectively known as Batten disease (BD), are a group of autosomal recessively inherited monogenic neurodegenerative diseases with shared aetiology. Mutations in 13 *CLN* genes have been reported to cause BD, with regional incidences varying from 1:12,500 live births in areas of Northern Europe to 1:50,000 in the U.S. [1]. Initial clinical presentation is almost exclusively due to visual failure and onset of epileptic seizures, progressing to cognitive and motor decline due to cerebral atrophy, ultimately resulting in mortality, often within the first decade. BD is clinically sub-categorized by age-of-onset into infantile, late infantile, juvenile, and rare adult-onset forms. Histologically, cerebral neurons exhibit enlarged lysosomes that accumulate autofluorescent lipofuscin, and neurodegeneration with associated neuroinflammation [1, 2].

Of the *CLN* genes affected by mutations in BD, four encode soluble lysosomal enzymes (CLN1/PPT1, CLN2/TPP1, CLN5 and CLN10/CTSD), where loss of protein function can rationally lead to substrate accumulation and lysosomal dysfunction stimulating neurodegeneration. Indeed, enzyme replacement therapy for CLN2 BD has proven clinically efficacious [3]. Mutations in *CLN5*, *CLN6, CLN7* and *CLN8* genes, result in the variant late infantile onset BD [1]. CLN5 is a lysosomal enzyme [4–6] and CLN6, CLN7 and CLN8 are all transmembrane proteins that map to the endolysosomal network. Mechanistically, CLN6 and CLN8 have been shown to coordinate the processing and vesicular transport of lysosomal enzymes, including CLN5, from the ER to the Golgi network prior to endosomal loading and transport to the lysosome [7–9]. Thus, there is an established mechanistic link between all variant late infantile BD forms, currently excepting CLN7.

CLN7 BD presents predominantly in late infancy but there are rare cases of diagnosis up to adulthood implying that there is phenotypic variability [10–12]. Moreover, the increased use of next generation genomic sequencing in diagnostics has revealed CLN7 genetic variants segregating with isolated retinopathies [13, 14], and the amyotrophic lateral sclerosis (ALS)/frontotemporal dementia (FTD) spectrum [15–17]. This implies shared pathogenic mechanism where CLN7 modulation contributes to an array or neurodegenerative diseases. There is currently no efficacious therapy for variant late infantile BD although gene therapies are showing potential in clinical trials. A more comprehensive understanding of the common mechanisms of pathobiology could help the development of unified therapeutic strategies, not only in BD but more widely across neurodegenerative disease.

The *CLN7* gene, located on human chromosome 4, encodes a 518-amino acid, ~57 kDa protein containing 12 transmembrane domains. Functionally, CLN7 has been described as a chloride channel localizing to the endolysosomal vesicular network [9, 18, 19]. However, there is strong evidence of CLN7 splice variants and post-translational modifications, including N-glycosylation at N371 and N376, and protease cleavage at the luminal Loop 9 (L9) of the transmembrane protein which could result in altered function [20–22]. Pathogenic nonsense, missense and splice site homozygote or compound heterozygote mutations have been characterized as causative in CLN7 BD (https://www.ucl.ac.uk/ncl-disease - updated July 2022) with the most common alleles being c.881 C > A, (p.Thr294Lys, T294K) and c.1393 C > T (p.Arg465Trp, R465W). Interestingly, 78% of identified CLN7 BD probands have at least one allele that is predicted to encode stable, but structurally compromised CLN7 protein with unknown consequences. Therefore, mechanistic studies conducted in the *Cln7* gene knockout mouse may not fully represent disease pathobiology in humans.

We have conducted a molecular deep phenotyping study in iPSC-derived neural progenitor cells (iNPC) from two CLN7 BD patients with T294K and R465W mutations and compared them with age-matched controls. Using molecular biology and metabolic assays, we have confirmed cellular defects in mitochondrial bioenergetics and autophagy in BD, with downstream effects consistent with lysosomal dysfunction, leading to apoptosis. Moreover, integrative comparisons of CLN7 and control transcriptomes, proteomes, and the CLN7 interactome indicate nuclear and endosomal defects independent of the lysosomal function, with elevated apoptotic signaling and apoptosis.

## Results

**Fig. 1:**
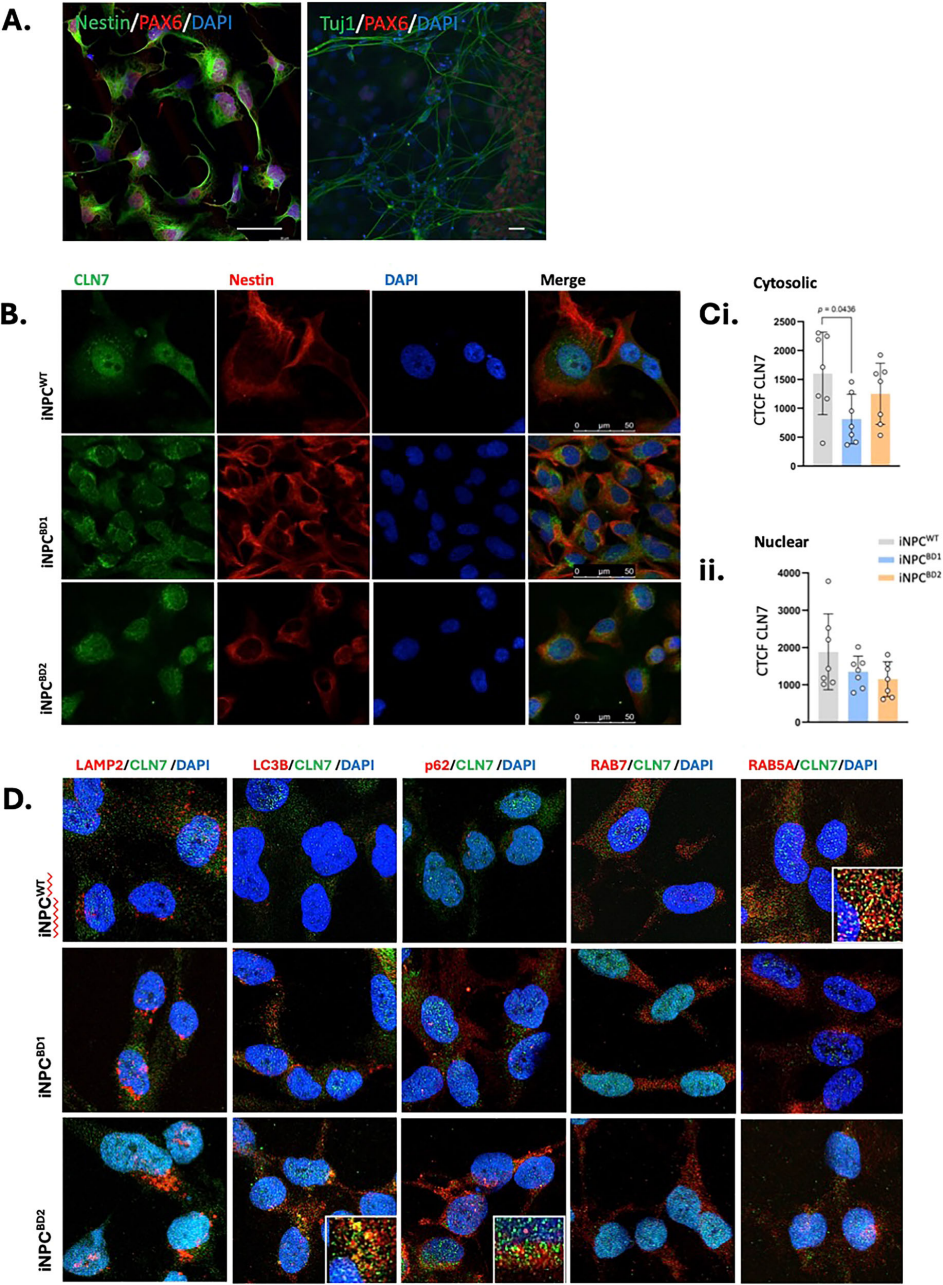
Intracellular localization of CLN7 in iNPC^WT^ and iNPC^BD^. **A** iPSC^BD1^ differentiation to Nestin^+^/PAX6^+^ iNPC and Tuj1^+^/PAX6^-^ iNeurons. Size bars = 50 µm (**B**) CLN7 ICC comparison of iNPC^WT^ and iNPCD^BD1&2^ with (**C**) quantitation of cytosolic and nuclear staining from at least 6 images containing multiple cells. Data is expressed as corrected total cell fluorescence (CTCF). Error bars indicate S.E.M., pairwise comparisons were made using a one-way ANOVA with post-hoc Tukey’s test. Size bar = 50 µm (**D**) Co-ICC with CLN7 and RAB5A, RABA7, p62, LC3 and LAMP2 comparison of iNPC^WT1^ and iNPC^BD1&2^. Size bar = 10 µm.

Clonal iPSC lines were derived from two variant late infantile BD patients; a female diagnosed at 2.5 years and genotyped with homozygous CLN7 c.881 C > A (p.T294K, hereafter referred to as BD1) and a male diagnosed at 4.5 years genotyped with homozygous CLN7 c.1393 C > T (R465W, hereafter referred to as BD2) (Table 1). We have previously shown that iPSC-derived neural progenitor cells (iNPC) from BD1 and BD2 present with lysosomal accumulation of globotriaosylceramide (Gb3) and subunit c of mitochondrial ATP synthase (SCMAS), as well as altered mitochondrial morphology, all indicative of BD neuronal phenotype [23, 24]. Here, we have conducted a comprehensive cellular phenotyping comparison of BD and age-matched control (WT) iNPC capable of specifying to cortical-like neurons (Fig. 1A).

**Table 1:** Genetic mutation, clinical presentation and progression for two BD patients designated Pa380 (BD1) and Pa474 (BD2).

Patient	Genotype	Sex	Age and Presentation	Clinical progression	Tissue pathology
Pa380 (BD1)	c.881 C > A p.T294K	Female	2.5 years with seizures	Mental regression, speech regression and motor impairment +3 years but with no myoclonous or visual failure.	*RL/FP complex CL on skin biopsy
Pa474 (BD2)	c.1393 C > T p.R465W	Male	4.5 years with motor impairment, mental & speech regression.	Motor impairment and myoclonous, but no ataxia or seizures	*FP, RL, CL on skin biopsy

### CLN7 mutation in BD alters its intracellular localization, where ectopic expression only partially recapitulates endogenous expression

CLN7 localized to cytosolic vesicles and the nucleus in iNPC by immunocytochemistry (ICC) using a polyclonal CLN7 antibody, but its relative distribution differed markedly between BD and WT (Fig. 1B). Quantitation of nuclear and cytosolic fluorescence per cell revealed a trend toward reduced CLN7 protein in both the nucleus and cytosol in BD which reached significance when comparing BD1 with WT iNPC (Fig. 1C). ICC co-localization of CLN7 protein was most frequent at RAB5A^+^ early endosomes in WT, whereas in BD it shifted to LC3B^+^/p62^+^ autophagosomes. LAMP2^+^ lysosomes were markedly enlarged in BD compared to WT but neither showed any overlap with CLN7 expression (Fig. 1D). Punctate cytosolic CLN7 immunostaining did overlap with endosomal markers in WT, and autophagic markers in BD, but in both instances there was also an abundance of non-overlapping signal. This implies there are other unidentified CLN7^+^ puncta in the cytosol of iNPC.

**Fig. 2:**
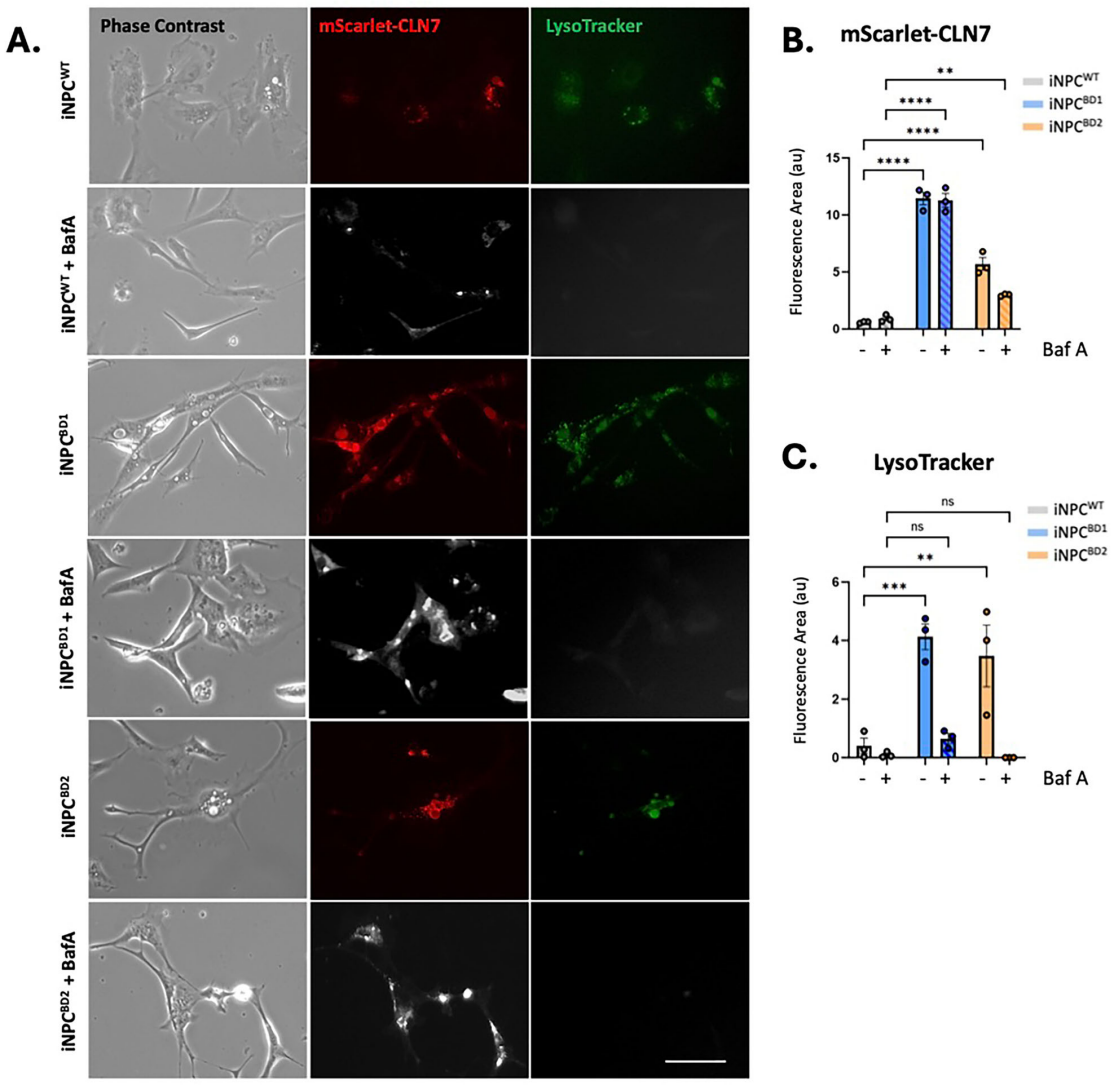
Intracellular localization of transgenically over-expressed CLN7. **A** iNPC^WT^, iNOC^BD1^ and iNPC^BD2^ were transduced with a lentivirus expressing an mScarlet-CLN7 fusion at 5 MOI and then co-stained with LysoTracker after 4-days following Bafilomycin A (10 nM) or DMSO carrier treatment for 12 h. The panel shows phase contrast, mScarlet-CLN7 (red) and LysoTracker (green) imaging of living cells. Size bar=30 µm. Corrected total cell fluorescence (CTCF) was quantified and compared for mScarlet-CLN7 (**B**) and LysoTracker (**C**) from a minimum of 10 captured images of 3 independent experiments. All data presented as mean ± SEM, *p ≤ 0.05, **≤0.01, ***≤0.001 and ****≤0.0001, (*p*-values in Table 8) Size bar = 30 µm.

Enlarged lipofuscin-containing lysosomes are a well characterized pathogenic phenotype of neural cells in BD, correlating with CLN7 loss of function [25]. However, Wang et al. have recently shown that exogenous over-expression of CLN7 in HEK293T cells results in the accumulation of CLN7^+^ mega-lysosomes, likely due to unchecked endolysosomal fusion [9]. We generated lentiviral vectors constitutively expressing mScarlet-CLN7 N-terminal fusion protein (Supplementary Fig. 1) then transduced SH-SY5Y neuroblastoma cells as well as WT and BD iNPC to evaluate CLN7 localization in living cells. mScarlet-CLN7 expression mapped almost exclusively to lysosomes, and we observed massively enlarged LysoTracker^+^/mScarlet-CLN7^+^ lysosomes in WT and BD iNPC and SH-SY5Y (Fig. 2A and Supplementary Fig. 2A, B). We frequently observed cells where a single lysosome occupied the majority of its cytosolic area. Lysotracker staining was retained within the lumens of these mega-lysosomes suggesting that they retain their ability to acidify this vast internal volume. Quantification showed that BD had a greater accumulation of both mScarlet^+^ and LysoTracker^+^ mega-lysosomes than WT iNPC (Fig. 2B, C). Over-expression of mScarlet-CLN7 induced mega-lysosomes, and cell death occurred in all transduced cells within 10 days of continued culture. We hypothesized that CLN7 is functionally involved in the endolysosomal fusion process mediated through vacuolar-type ATPase (vATPase). Bafilomycin A is a vATPase inhibitor and prevents lysosomal acidification and fusion with endosomes and autophagosomes [26]. Indeed, Baf A treatment of mScarlet-CLN7 transduced iNPC significantly reduced the emergence of mScarlet-CLN7^+^/Lysotracker^+^ lysosomes and reduced cell death (Fig. 2B, C & Supplementary Fig. 2A–B). Our data show that in WT iNPC, CLN7 is localized to endosomes, unidentified cytosolic puncta and the nucleus and this distribution is altered by pathogenic mutation. However exogenous expression of mScarlet-CLN7 fusion transgene resulted in almost exclusive lysosomal localization and apparently unchecked endolysosomal fusion. This implies that CLN7 localized to the nucleus and unidentified cytosolic puncta could be alternative isoforms, the result of alternative mRNA splicing.

### BD has a transcriptomic profile that correlates with neurodegenerative disease

**Fig. 3:**
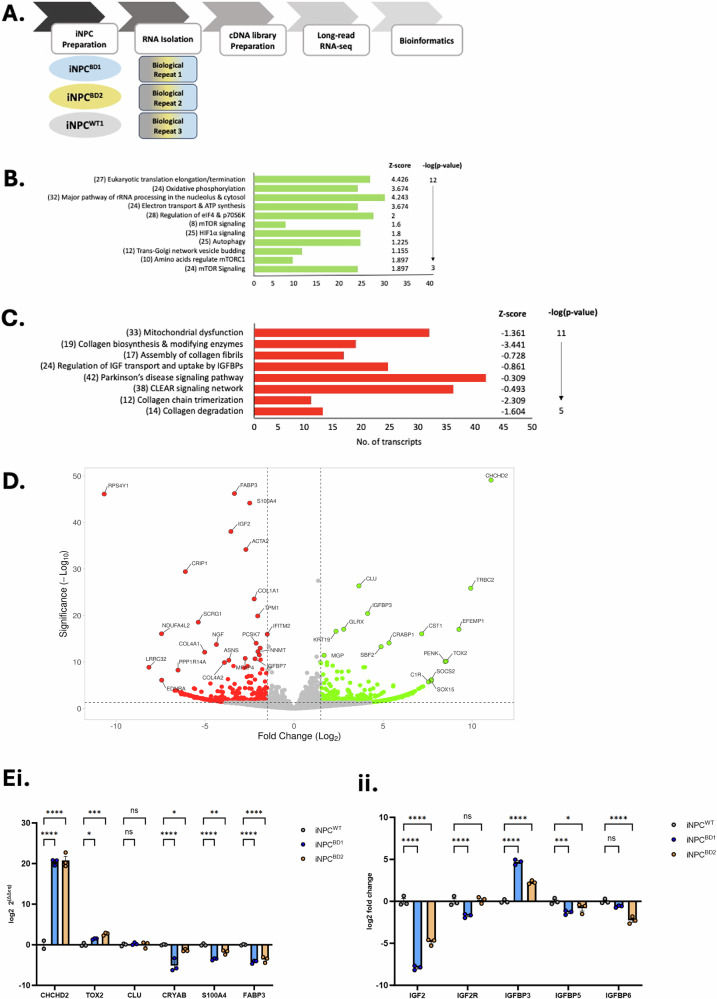
Unbiased transcriptomic comparison of iNPC^WT^ and iNPC^BD^. **A** Schematic workflow of long-read RNA sequencing. **Bi** Pathways including upregulated transcripts in iNPC^BD^ compared to iNPC^WT^ are ranked by z-score and -log(p-value). **Bii** p62 and the mTORC1 downstream target p70S6K are upregulated at the protein level on western blot. **C** Pathways including downregulated transcripts in iNPC^BD^ compared to iNPC^WT^ ranked by z-score and -log(p-value). **D** Volcano plot of transcript fold change (Log2) and significance (-Log10) for iNPC^BD^ versus iNPC^WT^. **Ei**, **ii** The expression of top decile upregulated and downregulated transcripts was evaluated in an independent experimental comparison of iNPC^BD^ versus iNPC^WT^ with 3 biological replicates. All data presented as mean ± SEM, *p ≤ 0.05, **≤0.01, ***≤0.001 and ****≤0.0001, (*p*-values in Table 8).

To evaluate the intracellular effects of pathogenic mutation on *CLN7* gene function we conducted an unbiased transcriptomic comparison of WT versus BD iNPC using long-read RNA sequencing (Fig. 3A). Consistent with our previous data in the *Cln7* knockout mouse cortical neurons (*Cln7*^ko^ CN) and those of others [23, 27], mTORC1 signaling, autophagy and mitochondrial oxidative phosphorylation (OXPHOS) were all upregulated processes in BD iNPC (Fig. 3B). Downregulated processes in BD included the CLEAR network, where TFEB can act through mTORC1 dependent and independent mechanisms (Fig. 3C) [24, 28, 29].

The top-ranking transcripts ranked by Manhattan distance included CHCHD2 (also known as MNRR1) and Clusterin (CLU) upregulation, with downregulation of FABP3 and S100A4, modulation, all having previously been associated with neurodegenerative disease (Fig. 3D, Tables 2 and 3); [30–33]. Interestingly, IGF2 was also in the top decile of downregulated and IGFBP3 an inhibitor of IGF2, in the top decile of upregulated transcripts. IGF2R, also known as the calcium independent mannose-6-phosphate receptor (CI-M6PR), transports lysosomal enzymes to the lysosome from the Golgi network [34], as well as being an active plasma membrane transporter in enzyme replacement therapies [3, 35]. We validated our RNA sequencing dataset using quantitative RT-PCR conducted on an independent BD versus WT comparison experiment (Fig. 3Ei–ii). Our qRT-PCR data fully reflected the transcriptomics, with the exception of CLU which was at undetectable levels in all samples. IGF2 and IGF2R were extensively downregulated, whereas IGFBP3 was concomitantly upregulated in both BD1 and BD2 compared to controls. We further interrogated their transcript variants in our long-read RNA sequencing datasets but there were no unexpected or unusual alterations in expression of predicted coding mRNAs (Supplementary Fig. 3).

**Table 2:** Top decile of upregulated transcripts ranked by Manhattan Distance of combined Fold change and significance in iNPC^BD^ versus iNPC^WT^.

Name	Fold change (log2)	Significance (p-value -Log10)	Manhattan distance
CHCHD2	11.06591415	49.10691918	60.17283332
TRBC2	9.924470423	25.85531867	35.77978909
CLU	3.643511368	26.37182294	30.0153343
EFEMP1	9.263777885	17.00989933	26.27367722
IGFBP3	4.134412383	20.42169324	24.55610562
CST1	7.169891738	16.05969888	23.22959062
GLRX	2.788128757	17.04304772	19.83117648
CRABP1	5.335897544	14.09487304	19.43077059
KRT19	2.359020279	16.62265506	18.98167534
TOX2	8.547148209	10.16845509	18.7156033
PENK	8.490096037	10.08345696	18.57355299
SBF2	4.890817753	13.29397505	18.18479281
SOCS2	7.712830571	6.200982101	13.91381267
SOX15	7.705149255	5.942586671	13.64773593
C1R	7.555636499	5.718129121	13.27376562
MGP	1.687985984	11.43220357	13.12018955

**Table 3:** Top decile of downregulated transcripts ranked by Manhattan Distance of combined Fold change and significance in iNPC^BD^ versus iNPC^WT^.

Name	Fold change (log2)	Significance (p-value -Log10)	Manhattan distance
RPS4Y1	−10.6664	46.11008	56.77644
FABP3	−3.35074	46.21365	49.56439
S100A4	−2.49071	44.16821	46.65892
IGF2	−3.54916	38.05636	41.60551
ACTA2	−2.70869	34.18325	36.89194
CRIP1	−6.10747	29.42996	35.53743
COL1A1	−2.23921	23.55051	25.78972
SCRG1	−5.38176	18.55866	23.94042
NDUFA4L2	−7.44547	16.07535	23.52083
TPM1	−2.0427	19.88903	21.93174
NGF	−4.36363	13.76337	18.127
IFITM2	−1.50874	15.96992	17.47866
COL4A1	−5.02298	12.10522	17.1282
LRRC32	−8.15554	8.860492	17.01603
PCSK7	−2.12919	14.03801	16.1672
SERPINH1	−1.90327	12.99543	14.89869
PPP1R14A	−6.52091	8.250159	14.77107
NNMT	−2.02805	12.23546	14.26351
ASNS	−3.66503	10.37215	14.03719
COL4A2	−3.91254	9.8833	13.79584
EDNRA	−7.43713	6.103111	13.54024
MFAP4	−2.74546	10.78025	13.52572
COL3A1	−1.95493	11.5307	13.48563
IGFBP7	−2.19482	10.68525	12.88007

A targeted evaluation showed that transcripts associated with vesicular transport, autophagy and the lysosome were upregulated in BD iNPC. The upregulated transcripts: AP1S3, AP1M2, CLVS1, GAK, STX17 and DNM2, all encode proteins that function in endosome-Golgi docking and transport. The autophagy initiating protein ATG9A transcript was upregulated, as were multiple lysosomal vacuolar ATPase H^+^ pump (vATPase) subunits ATP6V1G3, ATP6V1A and ATP6V0E2. Transcripts associated with the mitochondria were less generally deregulated (Supplementary Fig. 4). The transcript encoding the multi-functional mitochondrial protein CHCHD2 was the most significantly upregulated in both BD1 and BD2 compared to WT in iNPC. CHCHD2/MNRR1 determines cristae structure under normal conditions, but under stress conditions can interact with EIF2α at the ER and under hypoxic conditions has been shown to translocate to the nucleus and function as a transcription factor [36].

### Autophagic defects in BD are similar to those induced by Bafilomycin A in WT

**Fig. 4:**
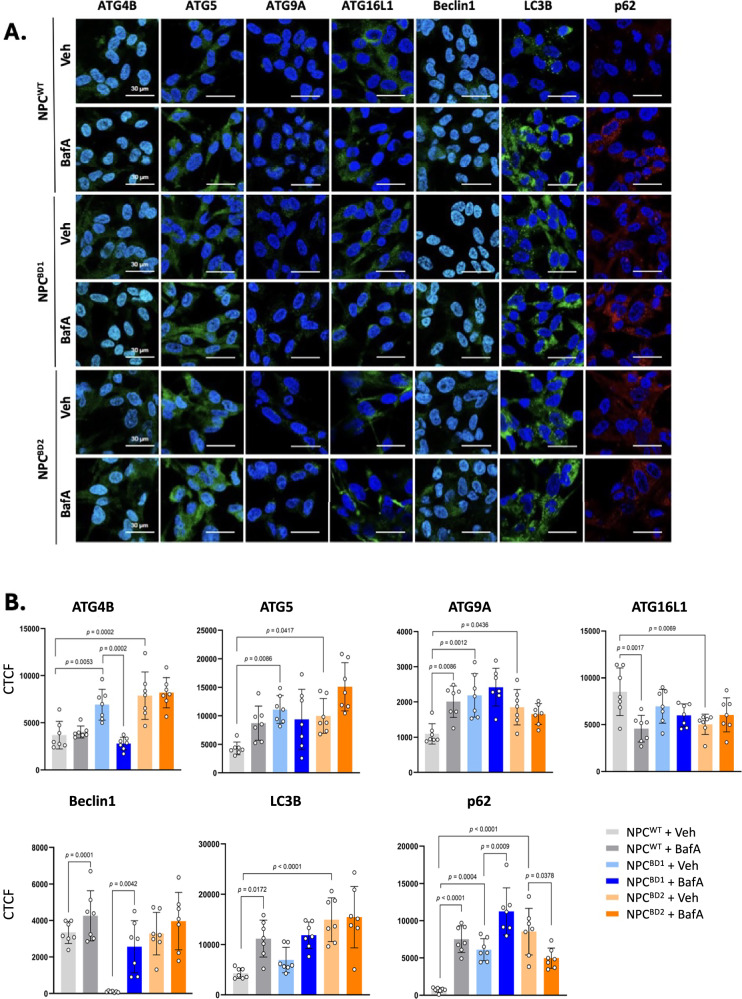
Evaluation of autophagy in iNPC^WT^ and iNPC^BD^. **A** Comparison of iNPC^WT1^ and iNPCD^BD1&2^ with established markers of autophagy by dual immunocytochemistry. Images are representative of 3 biological repeats. **B** Quantitation of autophagic marker staining was conducted from a minimum of 6 images containing multiple cells using ImageJ software. Data is expressed as corrected total cell fluorescence (CTCF). Error bars indicate S.E.M., pairwise comparisons were made using a one-way ANOVA with post-hoc Tukey’s test. All size bars = 30 µm.

Our unbiased transcriptomics and subsequent molecular validation recognized Golgi-endosome transport, autophagosome, lysosome and mitochondria as organelles affected in CLN7 BD. Having observed upregulation of transcripts encoding lysosomal vATPase we evaluated the effects of the vATPase inhibitor Bafilomycin A (Baf A) on autophagy and mitochondrial bioenergetics. Autophagy was assessed in BD iNPC using ICC for autophagy related proteins involved in autophagosome initiation (ATG9A), nucleation (Beclin1), conjugation (ATG4B, ATG5, ATG16L1) and maturation (LC3B, p62) in the presence or absence of Baf A (Fig. 4A) with ICC quantitated by corrected total cell fluorescence (CTCF; Fig. 4B). As expected, WT iNPC responded to Baf A treatment through the accumulation of autophagosomes due to inhibition of lysosomal fusion. However, ATG16L1 a multi-functional autophagy-related protein involved in autophagosome initiation, conjugation, and lysosome docking [37] was decreased in WT iNPC following Baf A treatment (Fig. 4B). This could be explained by a feedback response to failed lysosomal fusion and autophagosome accumulation due to Baf A treatment. Autophagy in BD iNPC under basal culture conditions was elevated to similar levels to WT following Baf A, and Baf A treatment did not have an additive effect. Consistent with upregulation of its transcript, ATG9A protein was significantly elevated in BD iNPC. There were however differences in BD1 and BD2 responses to Baf A which may relate to patient genotype and disease severity. In BD1 iNPC there were significant increases in Beclin1, LC3B and p62 protein whereas this was not evident in BD2. Although genotype-phenotype correlations cannot be concluded from two patients, BD1 was diagnosed earlier than BD2, and had more severe progression (Table 1). Overall, the BD cellular phenotype was that of elevated autophagy, consistent with an inability of autophagosomes to dock and fuse with lysosomes.

**Fig. 5:**
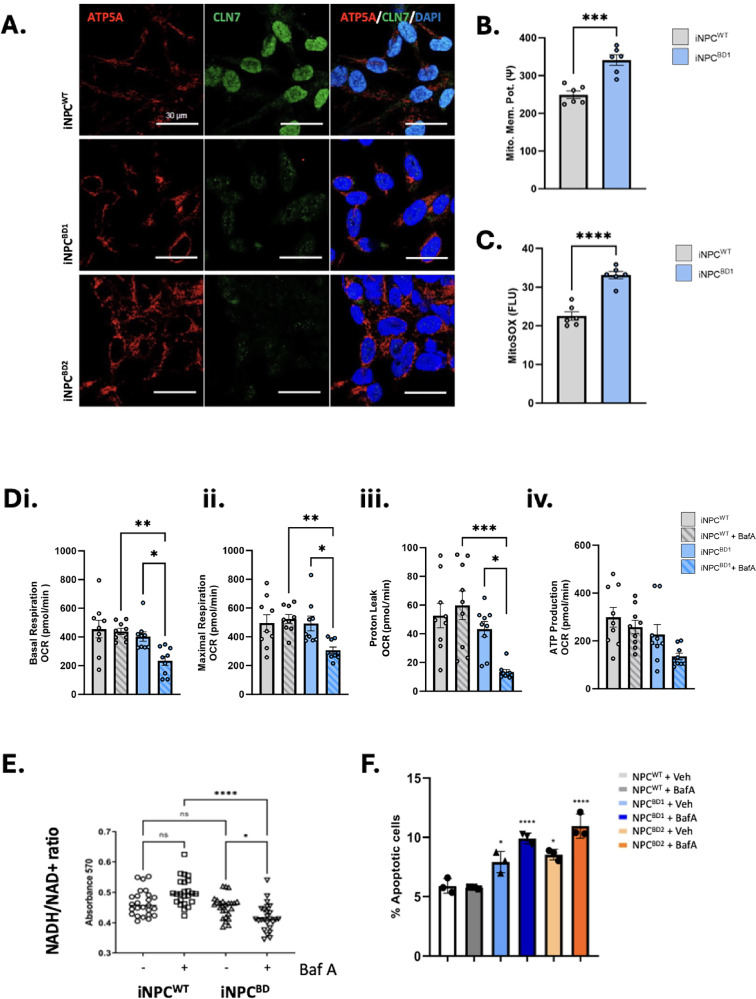
Altered mitochondrial bioenergetics leading to apoptosis in iNPC^BD^. **A** Immunocytochemistry comparison of iNPC^WT^ with iNPC^BD1^ and iNPC^BD2^ for the mitochondrial marker ATP5A. Images are representative of 3 independent experiments. Size-bar = 30 µm. Mitochondrial membrane potential was measured by TMRM (**B**, *n* = 6) and mitochondrial super oxide, quantified using MitoSOX (**C**, *n* = 6), are elevated in iNPC^BD1^ compared to iNPC^WT^. Mitochondrial respiration was assayed following 6 h of Baf A (10 nM) or DMSO vehicle using the Seahorse MitoStress test to quantify (**D**) (i) Basal and (ii) Maximal respiration (iii) Proteon Leak and (iv) ATP production and in iNPC^BD1^ compared to iNPC^WT1^ (*n* = 6) (*n* = 4) using the Seahorse MitoStress test. **E** Quantitation of NADH/NAD+ ratio by MTT assay in the presence and absence of Baf A in iNPC^BD1^ compared to iNPC^WT1^ (*n* = 6). **F** Apoptosis assay using Apotracker staining of gated viable cells that exclude eFluor-780 dye in the presence or absence of BafA in iNPC^BD1^ and iNPC^BD2^ compared to iNPC^WT1^ (*n* = 3). All data presented as mean ± SEM with pairwise comparisons using a one-tailed *t*-test. *p ≤ 0.05, **≤0.01, ***≤0.001 and ****≤0.0001, (*p*-values in Table 8).

Mitochondrial distribution was altered in BD iNPC under basal culture conditions with greater perinuclear localization of ATP5A^+^ mitochondria in BD iNPC (Fig. 5A). Indeed, BD iNPC also had significantly elevated levels of mitochondrial membrane potential (Fig. 5B) and mitochondrial reactive oxygen species (ROS; Fig. 5C). We further evaluated mitochondrial bioenergetics in the presence of Baf A to determine whether inhibition of lysosomal function was directly affecting mitochondrial function in BD. In WT iNPC there were no significant changes relating to mitochondrial oxidative phosphorylation (OXPHOS) with Baf A treatment. Interestingly, OXPHOS in BD iNPC was no different from WT iNPC, but Baf A treatment caused a significant reduction in basal and maximal respiration with a trend toward decreased ATP production. (Fig. 5Di–iv). This is mirrored by NADH/NAD+ ratio, indicative of a defective mitochondrial electron transport chain (Fig. 5E). We conclude that exacerbating lysosomal dysfunction alongside CLN7 loss of function compromises mitochondrial bioenergetics in BD INPC, thereby directly linking lysosomal inhibition with an acute and immediate defect in mitochondrial respiration. Mitochondrial membrane potential, elevated ROS and compromised bioenergetics are all widely reported drivers of apoptosis. Indeed, apoptosis was significantly elevated in both BD1 and BD2 compared to WT, and this is additively exacerbated by Baf A, but not WT iNPC (Fig. 5F). Moreover, we observed upregulation of apoptosis related transcripts in BD iNPC. We therefore conclude that basal apoptosis is elevated in BD and exacerbated by inhibition of lysosomal function, potentially through mitochondrial permeabilization and cytochrome c release.

### Golgi-endosome transport is upregulated, and RNA transport is downregulated in the BD proteome

**Fig. 6:**
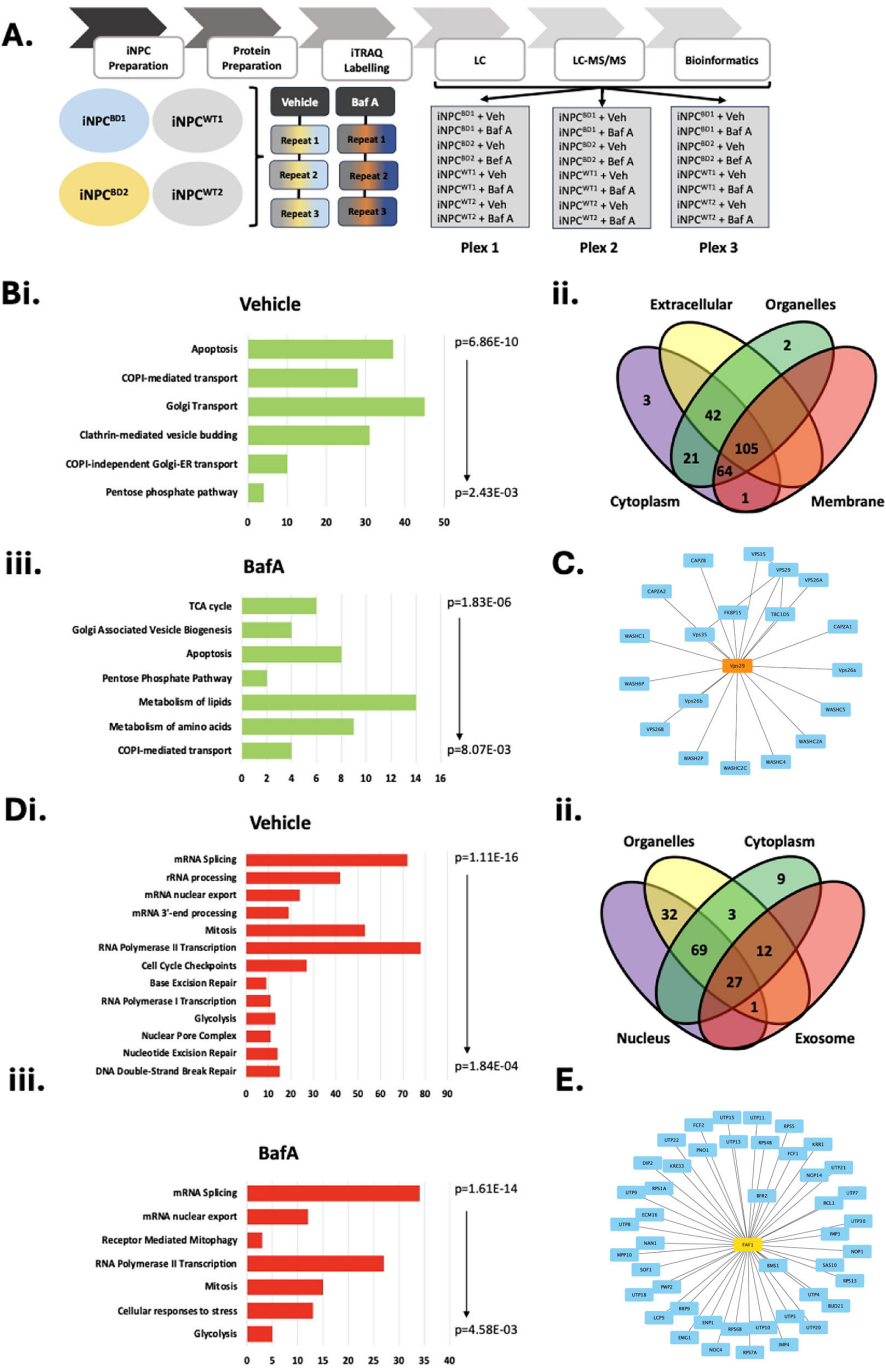
Unbiased global proteomics comparison of iNPC^BD^ to iNPC^WT^ with and without BafA treatment. **A** Schematic workflow of iTRAQ mass spectrometry, proteomic quantification and downstream bioinformatic analyses. Proteins with differential regulation of >2-fold with a *p* < 0.05 in iNPC^BD^ versus iNPC^WT^ were subjected to hierarchical Bayesian modeling then pathway changes identified using Cytoscape gProfiler through Reactome Pathway. **Bi** Upregulated protein pathways ranked by *p*-value and **Bii** separated by cellular localization. **Biii** Upregulated protein pathways ranked by p-value following Baf A (10 nM for 12 h) treatment. **C** A Minimal Essential Network identified in differentially upregulated proteins focussed around vesicular transport. **Di** Downregulated protein pathways ranked by p-value and **Dii** separated by cellular localization. **Diii** Downregulated protein pathways ranked by p-value following Baf A (10 nM for 12 h) treatment. **E** A Minimal Essential Network identified from differentially downregulated proteins focussed on RNA interactions.

Elevated autophagy, mitochondrial ROS and apoptosis are all broad markers of a cellular stress response. Such dynamic responses are often regulated at the post-transcriptional level, so we further conducted an unbiased proteomics matrix comparison of BD and WT iNPC. Experiments were performed in triplicate biological repeats for each individual (BD1, BD2, WT1 & WT2), and condition (DMSO or Baf A). Three separate mass spectrometry runs used three separate 8-plexes of isobaric tags for absolute and relative quantification (iTRAQ). A total of 24 iTRAQ tags were used to label the prepared peptides of the 24 samples so that each proteomic quantification run had the peptide sample of each cell sample with and without Baf A treatment (Fig. 6A). A comprehensive Bayesian analysis with pairwise comparisons was conducted with a total of 5619 proteins quantified and compared across all samples (Table 4). Differentially regulated proteins were analyzed for pathway alterations using Reactome Pathway application through Cytoscape and for cellular components using gProfiler through Cytoscape [38]. To identify key proteins that lie at a nexus of shared protein function, minimal essential networks (MEN) were built based on the public UniProt data base through Cytoscape. MEN contain the top 10% of BD associated proteins scored for connectivity and nexus network properties which represent the most functionally related regions of protein association models [39].

**Table 4:** Overview of upregulated and downregulated proteins from iTRAQ mass spectrometry within comparison groups of iNPC^BD^ versus iNPC^WT^ with and without Baf A treatment.

Comparison	Proteins with sig. *q*-value	Proteins upregulated (20% cut-off)	Proteins downregulated (20% cut- off)
**Control iNPC vs Control iNPC (Baf A** + **)**	0	-	-
BD iNPC vs BD iNPCs (Baf A + )	45	0	1
Control iNPC vs BD iNPC	1366	762	599
Control iNPC vs BD iNPC (Baf A + )	386	147	238

There was a remarkable division of cellular processes between those proteins upregulated and downregulated in BD versus WT iNPC under basal culture conditions. Upregulated processes in the BD proteomics mirrored the profile of upregulated transcripts, including apoptosis, but most notably vesicular transport across the ER and tarns-Golgi network (TGN), with proteins classifying with membrane and cytosolic localization (Fig. 6B i–ii). There is a subtle alteration in profile when comparing elevated proteins with and without Baf A treatment. In both conditions, ER/TGN vesicular transport and apoptosis are prevalent, however following Baf A treatment, there is an additional increase in transcripts associated with the TCA cycle and metabolism of amino acids and lipids (Fig. 6Biii). This is likely an integrated cellular stress response. Of the 103 proteins represented across MEN under basal culture conditions, 63 (61%) functionally map to vesicular transport, translational initiation/repression or mitochondria. Of these, 19 (18%) proteins mapped directly to an MEN node of ER/TGN/endosomal vesicular transport (Fig. 6C). This correlation unifies two unbiased evaluations of deregulation in BD iNPC corroborating our datasets and is consistent with the mechanistically characterized roles of CLN6 and CLN8 [7, 8].

Unexpectedly, depleted protein expression in BD iNPC is starkly centered on nuclear activities, including mRNA transcription, splicing and nuclear export, cell cycle and DNA damage response (Fig. 6Di–ii). Of 599 significantly downregulated proteins, 442 (74%) were functionally nuclear under basal culture conditions and 166 of 238 (70%) following Baf A treatment (Fig. 6Diii). The similarity of these profiles infers that the nuclear defect in BD iNPC was unaffected by lysosomal vATPase inhibition and could infer a nuclear function for CLN7 entirely separate from its emerging role in vesicular transport and membrane fusion at the lysosome. Of the proteins identified in the top decile of MEN, 49% were RNA associating or nuclear localizing proteins (Fig. 6E). Collectively, these data correlate well with an emerging hypothesis of lysosomal and nuclear defects combining to destabilize cellular metabolism, energy deficit under stress conditions, leading to mitochondrial-mediated apoptosis.

### The CLN7 interactome converges on RNA processing, translation initiation/repression and the stress granule

**Fig. 7:**
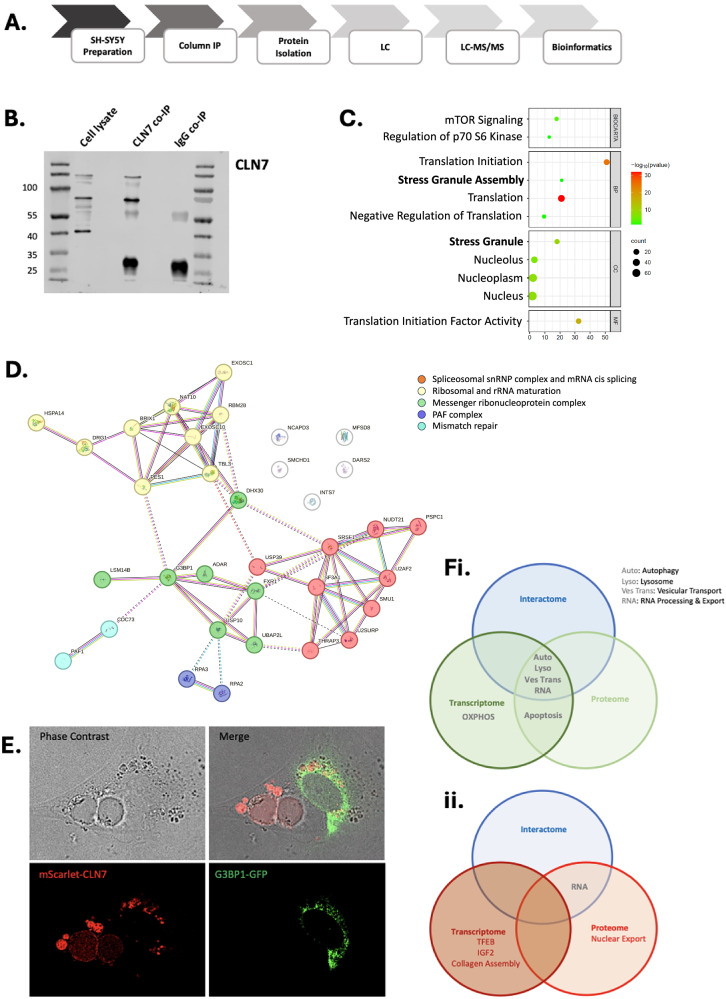
Identification of putative CLN7 associating proteins using co-immunoprecipitation mass spectrometry in SH-SY5Y neuroblastoma cells. **A** Schematic workflow of CLN7 co-immunoprecipitation, mass spectrometry and downstream bioinformatics. **B** CLN7 co-immunoprecipitation from SH-SY5Y cell lysates is specific in identifying CLN7 (57 kDa) and its N-glycosylated higher molecular weight forms on western blot alongside IgG light ( ~ 25 kDa) and heavy chain (50 kDa) bands. A lower molecular weight band in cell lysates likely represents a protease cleaved isoform. **C** Biocarta, Biological Process (BP), Cellular Component (CC) and Molecular Function (MF) displays from DAVID analysis. **D** STRING analysis of putative CLN7 interactors reveals associations with RNA processing and translation initiation. **E** co-localization of mScarlet-CLN7 fusion protein with the stress granule associating G3BP1-GFP fusion protein. Images are of selected dual transfected cells, but representative of multiple experiments. Size bar = 10 µm. Pathway terms that are shared between the interactome and the transcriptome, and proteome for upregulated (**Fi**) and downregulated (**Fii**) differential expression.

To correlate deregulated proteins in CLN7 BD with biological function, we conducted CLN7 co-immunoprecipitation (co-IP) pull down followed by mass spectrometry (Fig. 7A). We applied G-sepharose bead column co-IP using an anti-CLN7 polyclonal antibody and employing IgG as a non-specific binding control using SH-SY5Y neuroblastoma cells, as we were unable to yield sufficient protein from iNPC cultures. Immunoblots show that co-IP with CLN7 antibody captured protein consistent with CLN7 bands observed in whole cell lysates, which were absent in the IgG sample. Full-length CLN7 is predicted to encode a 57 kDa protein, consistent with the weaker lower band. The strongest band on the immunoblot is ~65-70 kDa which is consistent with a previously described glycosylated form [21] and a larger >120 kDa band could represent a dimer although this is not biologically proven. A band detected at ~25 kDa in both samples is consistent with auto detection of the light chain of the HRP tagged IgG secondary antibody (Fig. 7B).

Co-IP was performed on 2.5 mg protein lysate from 4 independent replicates for CLN7 and IgG control. A total of 186 interacting proteins were identified by mass spectrometry (FDR < 0.05, *p* < 0.01) with 65 registering positive hits in all 4 replicates and no positive hits in the controls. A combined interactor analysis employing Biocarta Biological Process (BP), Cellular Component (CC) and Molecular Function (MF) classifications showed mTOR signaling as a key component of the CLN7 interactome (Fig. 7C–D and Supplementary Fig. 5). Putative interactors included GATOR complex proteins DEPDC5, WDR24, WDR59 and SEH1L as well as the KICSTOR complex component SZT2 and the mTORC1 regulator TSC1 (Table 5). The largest and most significant cellular process within the interactome was ‘Translation Initiation’ (*p* = 2 × 10^−25^), but ‘Stress Granule Assembly’ (*p* = 1 × 10^−9^) was also a dominant cellular process, including the defining stress granule proteins G3BP1 and Caprin1. SH-SY5Y cells were grown under steady state culture conditions yet many of the putative CLN7 interactors related to the cellular stress response. SH-SY5Y cells were transduced with LNT-mScarlet-CLN7 and then transfected with a G3BP1-GFP fusion expression plasmid [40] to assess intracellular co-localization by live cell confocal microscopy. Consistent with previous experiments, within 4 days post-transduction mScarlet-CLN7 fluorescence localized with the formation of mega-lysosomes and in smaller perinuclear puncta. G3BP1-GFP expression was also perinuclear but non-overlapping with mScarelt-CLN7 (Fig. 7E). Our data shows CLN7^+^ puncta in close proximity but not overlapping with the stress granule marker G3BP1. Stress granules are not membrane-bound whereas CLN7 is confirmed as a transmembrane protein. This close association could represent an interface between stress granule and the endolysosomal network, but this remains to be experimentally validated.

**Table 5:** Alphabetic list of proteins identified as putative CLN7 interactors in SH-SY5Y cells.

CLN7 Interactome
AGO2, AKAP13, AKAP9, ANK2, APC, ARHGEF11, ARHGEF7, ASCC1, ASCC3, ATM, ATR, ATXN2L
BCLAF1, BCR
CACYBP, CAMK4, CAMSAP3, CAPRIN1, CCDC85C, CDC73, CDK5RAP2, CENTG3, CNOT3
DARS2, DCAF1, DCAF7, DDX21, DDX23, DDX27, DDX41, DDX46, DDX56, DECR1, DEPDC5, DHX15, DHX29, DHX30, DHX57, DKFZp, DNAJC2, DOCK6, DRG1, DYRK1A
EIF3F, EIF3H, EIF3I, EIF3M, EIF3S1, EIF3S8, EIF4G1, EXOSC10, EXOSC2, EXOSC6
FAM120C, FAM98A, FMR1, FOCAD, FXR1, FXR2
G3BP1, G3BP2, GIT2, GNL3, GTPBP1, GTPBP4
H1FX, HAUS6, HBB, hCG, HDLBP, HEATR1, HERC2, HERC4, HPS5
IFT140, INTS10, INTS13, INTS2, INTS3, INTS4, INTS5, INTS6, INTS8
KIF14, KIF21A, KIF7
LARP1, LRCH2, LRCH3, LZTS2
MAPRE2, MCM6, MED23, MICALL2, MSH6, MTREX, MYBBP1A, MYCBP2, MYEF2, MYO5A, MYO6
NAT10, NCAPD3, NF1, NOP2, NPM1, NUDT21, NUFIP2
PABPC4, PAF1, PAN3, PDE3A, PDS5A, PES1, PIK3R4, PRKACA, PRKAR2B, PRKRA, PRPF19, PRPF6
RAD50, RAE1, RALGAPB, RBM20, RBM25, RBM26, RBM28, RICTOR, RPA1, RPA3, RPL29, RPL36AL, RPS27, RSL1D1
SACS, SART1, SBF1, SEH1L, SF3A1, SF3B1, SFRS2, SIPA1L3, SMARCAL1, SMC6, SMCHD1, SMU1, SNRPD3, SNX3, SPATA5, SPATA5L1, SPT5, SRSF1, SZT2
TBL3, TCF4, TJP1, TLK2, TOP3B, TPM1, TRIM25, TSC1, TSR1, TTC37, TUBG1
U2AF1L5, U2AF2, U2SURP, UBAP2L, USP10, USP39, UTP18
VPS16, VPS18
WDR24, WDR3, WDR35, WDR36, WDR5, WDR59, WDR61
YTHDC2

Consistent with the proteomic nuclear phenotype in BD there was also an enrichment of nuclear proteins, particularly those that interact with RNA, including RNA helicases (DDX23, DHX29, DHX30, and DHX57), integrator complex proteins (INTS3, INTS4) and RNA stability (CDC73, GTPB1, GTPB4). DNA damage/repair pathways were also strongly represented (ASCC1, ASCC3, BCLAF1, RPA1, RPA3) (Fig. 7Fi–ii, Table 6). The CLN7 interactome segregates with endolysosomal and perinuclear protein binding partners that relate to RNA processing and translation initiation/repression at the stress granule. This segregation infers multiple functions for CLN7 that converge around the cellular stress response. Overall, our data show that pathogenic mutation results in the loss of multiple CLN7 functions leading to apoptosis in iNPC. CLN7 protein functions center around cellular stress response including endolysosomal processing and RNA translation initiation/repression at the stress granule. Notably, over-expression of CLN7 results in unchecked endolysosomal fusion that results in cell death.

**Table 6:** Matrix lists of transcript and proteins showing overlap between upregulated and downregulated transcriptome and proteome in iNPC as well as the CLN7 interactome in SH-SY5Y cells.

	Upregulated	Downregulated
Transcriptome & Interactome	AKAP9, AKAP13, ATXN2, AUTS2, DEPDC5, DHX30, EXOSC5, FMR1, FRYL, HPS5, INTS10, NHSL2, TRRAP	ANK2, IFT140, INTS7, MYCBP2, TSC1
Proteome & Interactome	AKAP9, ANK2, ARFGEF2, ARHGEF7, DECR1, LRCH3, RNF213, TJP1, VPS18	ADAR, BRIX1, CDC73, DARS2, DHX30, DRG1, EXOSC1, EXOSC10, FXR1, G3BP1, HSPA14, INTS7, LSM14B, NAT10, NCAPD3, NUDT21, PAF1, PES1, PSPC1, RBM28, RPA2, RPA3, SF3A1, SMCHD1, SMU1, SRSF1, TBL3, THRAP3, U2AF2, U2SURP, UBAP2L, USP10, USP39
Transcriptome & Proteome	AKAP9, ARFGEF1, ARHGEF12, ATP6V1A, CASP3, CC2D1B, CD47, DNM2, DST, EXTL3, GNAI1, HOMER3, HTT, KIF13B, KLC4, MTCL1, PAXIP1, PDLIM4, PIKFYVE, PRKD2, PSMD5, RASSF8, SCFD2, SYNM, TANC1, TJP2, TOM1, UDE3A, USO1, VPS13C, WASF1	ACADM, CYB5R2, FABP5, INTS7, LBR, PHGDH, PTBP2, VCAM1, VRK1

## Discussion

BD biology is complex, where biallelic pathogenic mutations in up to 13 genes result in neurodegeneration with varying onset and severity. Biallelic mutations in the *CLN7* gene cause the variant late infantile NCL (BD) as do biallelic mutations in *CLN5*, *CLN6* and *CLN8* genes [1]. Furthermore, there is an emerging genotype-phenotype correlation where some *CLN7* gene mutations associate with disease severity, although the rarity of vLINCL precludes any firm conclusions [41]. vLINCL most often presents clinically with visual failure, manifested through macular dystrophy, and interestingly, biallelic and monoallelic *CLN7* gene variants have also been characterized in patients with isolated macular dystrophy and retinopathy [13, 14]. Moreover, *CLN7* gene variants have also been shown to segregate with other neurodegenerative disease, mapping to the amyotrophic lateral sclerosis/frontotemporal dementia spectrum (ALS/FTD) [15–17]. These clinical commonalities infer shared molecular mechanisms contributing to neurodegeneration.

Our data indicate that CLN7 protein has at least two apparently independent cellular functions; the first enabling endosome docking and/or fusion at the lysosome and potentially at the TGN. The second apparently separate function facilitating RNA transport from the nucleus and influencing translation initiation/repression at the ribosome. We detect endogenous CLN7 at nuclei and early endosomes in WT but there is a shift from endosome to autophagosome localization in BD iNPC. In our experiments CLN7 is barely detected at WT or enlarged BD iNPC lysosomes which no longer present as acidified using LysoTracker staining. A number of mTOR signaling proteins (TSC1, SZT2, DEPDC5, WDR59, TJP1) are represented in the CLN7 interactome, suggesting close association with lysosomal tethering and signaling, whereas components of TFEB signaling are downregulated in the proteome. When exogenously expressed, CLN7 localizes exclusively to lysosomes which subsequently become massively enlarged. These mega-lysosomes are positive for luminal LysoTracker staining meaning they are acidified, a phenotype that appears vATPase-dependent due to rescue with Baf A treatment. Our data in iNPC aligns strongly with that of Wang et al. who observed mega-lysosomes when exogenously expressing CLN7 in 293T cells and that CLN7^T294K^ mutation reversed the mega-lysosome phenotype [9]. vATPase activity and lysosomal acidification is regulated by CLC family of passive Cl^-^ channels [42]. CLN7 is an outward-rectifying Cl^-^ channel, which could influence the homeostatic vATPase/CLC balance when accumulating at lysosomal membranes due to exogenous supraphysiological expression. This accumulation may result in unchecked HOPS/SNARE-mediated endolysosomal fusion. These observations were made using exogenously expressed mScarlet-CLN7 fusion protein. The N-terminal fusion with a fluorescent marker could have unanticipated consequences, but the observed endolysosomal localization appears to correlate with endogenous localization and N-terminal fusions/tags been used effectively by others studying CLN7 biology [9, 43]. We acknowledge that transgenic over-expression may not represent endogenous CLN7 function, but this is conversely extremely important in evaluating the effects of gene therapy where supraphysiological CLN7 expression is often unavoidable.

The CLN7 interactome includes the core HOPS subunit VPS18, but also VPS26A/B, VPS29 and VPS35 proteins are increased in BD iNPC, all of which are components of the retromer complex which recycles proteins from endosomes to the Golgi and where gene mutations have previously been associated to neurodegenerative disease [44]. Retrograde vesicular transport from endosome to Golgi and Golgi to ER are the most significantly upregulated protein classifications in the BD iNPC proteome. Our data converge on a functional role for CLN7 in endolysosomal fusion whereby loss of function could result in failed fusion, creating a feedback loop of elevated retromer-mediated recycling from late endosomes to the TGN.

The calcium-independent mannose-6-phosphate (M6P) receptor IGF2R is responsible for the vesicular transport of over 70 hydrolases with M6P domains from TGN and plasma membrane to the lysosome, enabling enzyme replacement therapies for BD [3] and other lysosomal storage diseases. Importantly, CLN3 has recently been linked to IGF2R activity through autophagic and endolysosomal transport [45]. CLN3 and CLN7 are structurally similar transmembrane proteins, and mutations in these genes cause BD suggesting that they may share pathogenic mechanisms. IGF2R is recycled from endosomes to the TGN by retromer complexes and is also increased in our BD iNPC. IGF2R M6P-binding activity is competitively inhibited by circulating IGF2, which is itself inhibited by IGFBP3. IGF2 is in the top decile of downregulated transcripts, and the IGF2 inhibitor IGFBP3 is in the top decile of upregulated transcripts in BD iNPC. Therefore, circulating IGF2/IGFBP3 could represent strong candidates as circulating biomarkers for identifying BD.

Autophagy shares much of the lysosomal fusion machinery with endosomes, and our data is consistent with CLN7 mutation also resulting in failed autophagosome-lysosome fusion. Interestingly, we do not detect CLN7 in WT autophagosomes, but do observe occasional CLN7^+^ autophagosomes in BD iNPC. This could be the result of defective endosomal protein content being diverted to the autophagic degradation pathway. However, this hypothesis would require experimental validation. It was notable that autophagy marker quantity and localization in BD mirrored WT iNPC treated with Baf A. This is consistent with a lysosomal docking/fusion defect caused by CLN7 loss of function. Further evidence of a negative feedback loop caused by lysosomal stress is that cytosolic ATG5 is significantly elevated in BD iNPC and has recently been described as an autophagosome-independent feedback inhibitor of the retromer complex [46].

A consequence of lysosomal dysfunction is the breakdown of mitophagy in BD [23, 47], other lysosomal storage diseases (LSD) [48], as well as Alzheimer’s [49] and Parkinson’s disease [50]. Mitochondria are constantly degraded by mitophagy in neurons with high metabolic requirements, closely matched by mitochondrial biogenesis. We report increases in ATP5A^+^ mitochondria, mitochondrial membrane potential and ROS, all indicative of the accumulation of dysfunctional mitochondria. Mitochondrial OXPHOS was decreased following 6 h of Baf A treatment implying an acute inhibition unlikely to be the result of impaired mitophagy. It is plausible that this could be due to alterations in cytosolic Ca^2+^ concentrations due to inhibition of lysosomal acidification [51] and potentially endoplasmic reticulum localized SERCA [26] having a secondary effect on mitochondrial function but this remains to be scientifically validated.

The BD upregulated proteome included DIBALO and cytochrome c, who’s combined release from mitochondria with elevated membrane potential precede apoptosis through caspase-3 cleavage [52]. Caspase-3 was also upregulated in the BD transcriptome and proteome. BD iNPC contained significantly more apoptotic cells than WT under basal culture conditions and this was further exacerbated by Baf A in BD but not in WT iNPC. These findings suggest that mitochondrial permeabilization, although a secondary effect of BD, could be the primary driver of apoptosis and thus neurodegeneration. However, dysfunctional lysosomes can also become leaky, leading to elevated apoptosis. Recent studies show that leaky lysosomes in BD, other LSDs and PD release mitochondrial double stranded DNA (dsDNA) and initiate apoptosis through cGAS-STING innate immune response [50, 53, 54]. The cGAS-STING1 activation of interferon due to the cytosolic presence of dsDNA is mediated through IRF3 which is significantly upregulated in the BD proteome. Interestingly, ATG5/LC3, both significantly upregulated in BD iNPC, interact with cGAS-STING to induce autophagosome formation through COP-II mediated ER to Golgi transport [55, 56]. Our data are consistent with the combined cytosolic release of mitochondrial DIABLO/cytochrome c and lysosomal dsDNA contributing to elevated apoptosis in BD iNPC, potentially resulting in neurodegeneration.

The most profound and unexpected discovery from our study was that we detected the majority of CLN7 immunolocalization in the nucleus of iNPC. CLN7 missense mutations have also implicated in ALS/FTD [15–17, 57] where Geier and co-workers detected nuclear localization of CLN7 in neurons of the middle frontal gyrus and cultured dermal fibroblasts from FTD patients with CLN7 gene variants [15]. Processes involved multiple nuclear functions, including mitosis, DNA repair, RNA transcription, processing, splicing, and nuclear export were significantly downregulated in BD iNPC at the transcriptome and proteome level. Interestingly, multiple RNA interacting proteins were evident in the interactome. The polyclonal CLN7 antibody used for IP also detects its nuclear localization suggesting that CLN7 may have a trans-nuclear function, facilitating the transport of RNA from nucleus to ribosomes. Pathogenic mutations in CLN7 may result in a more generalized impairment of nuclear function. Notably, *AKAP9* is the only gene that has upregulated transcript and protein in BD and is also identified as a putative CLN7 interactor. Functionally, it is an adapter protein that forms a membrane contact link between Golgi and nucleus [58]. AKAP9 interaction could potentially provide a link between vesicular protein transport to the lysosome and nucleus. The proteomic data showing a decrease in nuclear proteins is unbiased and representative of CLN7 loss of function, but we acknowledge that the CLN7 co-immunoprecipitation only detects putative interactors involved in RNA transport which would require independent validation through proximity ligation assays such as Bio-ID.

Finally, our multi-omics data analysis of BD in iNPC converges on the stress granule, a non-membrane bound cytosolic nucleate, containing mRNAs and RNA binding proteins that act to regulate mRNA translation initiation/repression at the ribosome. From 186 putative CLN7 proximal association proteins, 55 interact directly with RNA, or are components of RNA binding complexes, both in the nucleus and cytosol. Nuclear RNA associating proteins include: 7 INTS integrator complex proteins that regulate polymerase II-mediated transcription, 5 DHX and DDX RNA helicases, and 6 EXOSC proteins involved in exosome-mediated RNA degradation. Cytosolic RNA interactions centered around the stress granule with the core stress granule proteins G3BP1 and Caprin1 being two of the most significant hits in the CLN7 proximal association interactome. In total, 16 other stress granule associated proteins appeared as putative proximal associators with CLN7, including FXR1, ATXN2, and 6 elongation initiation factors (EIF). Stress granules have recently been implicated in Charcot-Marie-Tooth type 2 neuropathies [59] and most pertinently CLN3 putative interactors [60]. As with our study on CLN7, Relton et al. show that CLN3 also immunoprecipitates with the two key stress granule proteins G3BP1 and Caprin1. Intriguingly, Liao et al. recently described a novel mechanism of RNA transport in neurons whereby ANXA11 acts as a molecular tether to facilitate the attachment of RNA-containing granules to lysosomes, aiding axonal transport and regional protein translation [61]. Our confocal imaging shows CLN7 perinuclear puncta in close but non-overlapping proximity to G3BP1 nucleates. This could be further evaluated using Bio-ID. Such proximity would be consistent with CLN7 playing a role in this close association that becomes destabilized in neurodegenerative disease. Collectively, these studies and our own suggest that the structurally similar CLN7 and CLN3 proteins may share dual functions in endosome and autophagosome fusion with lysosomes, as well as a stress granule interaction that interfaces with lysosomes and respond to lysosomal stress.

In conclusion, our data indicate for the first time that CLN7 has multiple functional roles that link cellular stress responses at the lysosome, mitochondria and nucleus, all membrane-bound organelles. Multi-omics converge on CLN7 interactions and function in endosomal transport to the lysosome where loss of function results in disruption to nuclear activities, notably RNA export, and apoptosis. This study provides new molecular insights into how pathogenic mutations in the *CLN7* gene results in neurodegeneration in BD. Moreover, CLN7 gene variants have been identified in ALS/FTD and macular degeneration implying CLN7-associated intracellular functions that could represent common and convergent therapeutic targets for neurodegeneration. Importantly, we identify IGF2 and IGFBP3 as potential circulating biomarkers of CLN7 that could aid diagnostics in the future.

## Materials and Methods

### Culture of human hiPSC

iPSC lines were previously generated as described using episomal doggybone DNA vectors [62] using dermal fibroblasts held by the UCL NCL database and cell repository (https://www.ucl.ac.uk/ncl-disease/), donated by two CLN7 patients (BD1; homozygous for c.881 C > A, p.Thr294Lys and BD2; homozygous for c.1393 C > T, p.Arg465Trp) and two age-matched controls (PromoCell). All iPSC were maintained on inactivated MEFs feeder layer and transitioned to feeder-free in preparation for neural specification. iPSC maintenance medium is composed of DMEM/F-12 (Gibco™), 2% L-Glutamine, 20% Knockout™ Serum Replacement (Gibco™), 1× MEM Non-Essential Amino Acids Solution (100×) (Gibco™), 1× N-2 Supplement (100×) (Gibco™), 0.1 mM β-mercaptoethanol (50 mM), 10 ng/ml Recombinant Human FGF-basic (PrepoTech®), 1% Pen/Strep and 0.2% MycoZap™ prophylactic (Lonza). Essential 8™ Medium (Gibco™) was prepared for feeder-free iPSCs culture on Matrigel® Matrix by addition of the Essential 8™ Supplement (50×) to the 500 mL Essential 8™ Basal Medium according to the manufacturer’s instructions and 1% Pen/Strep.

### Neural specification of iPSC

Plates and flasks for feeder-free iPSCs, neural stem cells (NSCs) and iNPCs culture were previously coated with Matrigel® Matrix (Corning®). For iPSCs culture on feeders, mitotically inactivated MEFs were plated on previously coated wells with autoclaved 0.1% gelatin. Neural Induction Medium (NIM) was prepared and used for culturing neurospheres and NSCs, and it is composed of DMEM/F-12 (Gibco ™), 1× MEM Non-Essential Amino Acids Solution (100×) (Gibco™), 1× N-2 Supplement (100×) (Gibco™), 20 ng/ml Recombinant Human FGF-basic, 0.1% Heparin, 1% Pen/Strep and 0.2% MycoZap™ prophylactic (Lonza). Neural Expansion Medium (NEM) was employed for culturing NPCs, and it is formed of DMEM/F-12 (Gibco ™), 1× MEM Non-Essential Amino Acids Solution (100×) (Gibco™), 1× N-2 Supplement (100×) (Gibco™), 1× B-27™ Supplement (50×) (Gibco ™), 20 ng/ml Recombinant Human FGF-basic, 0.1% Heparin, 1% Pen/Strep and 0.2% MycoZap™ prophylactic (Lonza).

iPSCs colonies were cultured on Matrigel® Matrix on 6-well plates at 80% confluency. Cells were washed with PBS and dissociated with TrypLE™ Express (Gibco™). They were centrifuged at 107 × g for 8 min and resuspended in Essential 8™ supplemented medium. Cells were counted using a Neubauer chamber and 7.5 × 10^3^ cells per well were plated on V-Shaped Bottom plates (low-attachment 96-well plates) in 100 μl of Neural Induction Medium (NIM) and ROCK inhibitor (Y-27632 dihydrochloride, 1254, Biotechne®) (used at 1:1000). Half of the medium (50 μl) was changed every day during 5 days after cell plating to allow cells to form spheroids. After 5 days of medium change, 10-15 spheroids per well were plated in 6-well plates previously coated with Matrigel® Matrix (Corning®). NSCs (neural rosettes and neural tubes) were dissociated with TrypLE™ Express and centrifuged at 107 × g for 8 min for neural differentiation. The cell pellet was resuspended in Neural Expansion Medium (NEM) and cells were plated on new 6-well plates coated with Matrigel® Matrix to generate NPCs which grow as single cells.

### SH-SY5Y culture

SH-SY5Y cells were cultured on complete DMEM/F-12 (Gibco™) (10% Fetal Bovine Serum, 2% L-Glutamine, 1% Penicillin/Streptomycin and 0.2% MycoZap™).

### Plasmids and transfections

Plasmid; pEGFPC1-CLN7 was a kind gift from Dr. Stephan Storch (University of Hamburg) and have been previously described Steenhuis et al. [21] The pcDNA3.4-e7/8CLN7 plasmid was de novo synthesized by GeneArt (Thermo Fisher); iNPC, MEFs, primary cortical neurons and SH-SY5Y cells were transfected using Lipofectamine LTX reagent (Thermo Fisher) according to the manufacturer’s protocol with 2 µg DNA per 9.6 cm^2^ well and cells analyzed 24–72 h later.

### Bafilomycin A treatment

iNPC and primary cortical neurons were treated for 6 h with 100 nM bafilomycin A (1334, Tocris) for the bioenergetics and immunocytochemistry analyses.

### Bioenergetics analysis

Oxygen consumption rate (OCR) of iNPCs and *Cln7*^*Δex2*^ mouse cortical neurons were measured in real-time in an XFe24 Extracellular Flux Analyzer (Seahorse Bioscience; Seahorse Wave Desktop software 2.6.1.56). The instrument measures the extracellular flux changes of oxygen in the medium surrounding the cells seeded in XFe24-well plates. Assays were performed 1 to 7 days after cell plating. Regular cell medium was removed, and cells were washed twice with DMEM running medium (XF assay modified supplemented with 5 mM glucose, 2 mM L-glutamine, 1 mM sodium pyruvate, 5 mM HEPES, pH 7.4) and incubated at 37 °C without CO_2_ for 30 min to allow cells to pre-equilibrate with the assay medium. Oligomycin, FCCP or antimycin/rotenone diluted in DMEM running medium were loaded into port-A, port-B or port-C, respectively. Final concentrations in XFe24 cell culture microplates were 1 μM oligomycin, 2 μM FCCP and 2.5 μM antimycin and 1.25 μM rotenone. The sequence of measurements was as follows, unless otherwise described. Basal level of OCR was acquired in 3 consecutive determinations, and thereafter oligomycin was injected and mixed for 3 min before the OCR was acquired in 3 consecutive determinations. The same procedure was followed for FCCP and antimycin/rotenone. All determinations were normalized to the basal OCR obtained in the corresponding well. Three to five replicas were determined for each sample obtained from the number of independent cell culture preparations indicated in the figure legends. The non-mitochondrial OCR was obtained after antimycin/rotenone injection, and the maximal respiration was obtained after FCCP injection minus that of the non-mitochondrial OCR. ATP production was calculated by subtracting, to the last OCR value before oligomycin injection, that obtained after oligomycin injection.

### Cell immunocytochemistry

iPSCs and iNPCs cultured on 12-well plates or on Nunc Lab Tek chamber slides (8 wells) were washed twice with PBS and fixed with 100% ice-cold methanol for 5 min (on ice or at −20 °C). Cells were washed again with PBS three times. Cells were blocked with blocking solution (2% BSA in PBS, 1% goat serum, 0.1% Triton X-100) for 30 min on the rocker. Primary antibodies were prepared in blocking solution at a 1:100 dilution. Cells were incubated with primary antibodies for 2 h at room temperature and washed three times with PBS. Secondary antibodies (A110088, Alexa Fluor ™ 488 goat anti-rabbit; A11055, Alexa Fluor ™ 488 donkey anti-goat; A11004, Alexa Fluor™ 568 goat anti-mouse; and A11011, Alexa Fluor ™ 568 goat anti-rabbit) were prepared at 1:500 dilution in blocking solution and cells were incubated with them for 1 h at room temperature. Cells were washed three times with PBS. To stain cell nuclei, 1 mg/ml DAPI solution (D9542, Sigma-Aldrich) used at 1:1000 in PBS was added to the cells for 15–30 min or a drop of the DAPI Mounting (Vectashield®) solution was added to each well. In the first case, cells were washed three times with PBS, and they were ready to image after the addition of PBS to each well. In the second case, cells were incubated at 4 °C overnight before imaging them. Cells were imaged using the Leica 6000CTR Live cell imaging fluorescent microscope (for plates) and the Zeiss Imager M2 microscope (for slides). Details of antibodies can be found listed in Table 7.

**Table 7:** List of antibodies.

Human Antibody	Raised Species	Company	Catalogue	Western Dil.	ICC Dil.
CLN7^1-37aa^	Rabbit polyclonal	Invitrogen	PA5-60832	1:500	1:100
CLN7^351-421aa^	Rabbit polyclonal	Proteintech	24298-1-AP	1:1000	
SOX2	Rabbit monoclonal	Abcam	Ab97959		1:200
βIII-tubulin	Mouse monoclonal	R&D Systems	MAB1195		1:200
Nestin	Mouse monoclonal	R&D Systems	MAB1259		1:200
LAMP1	Rabbit polyclonal	AbCam	Ab24170		1:200
LAMP2	Rabbit monoclonal	Santa Cruz	Sc-18822		1:200
p62	Mouse monoclonal	AbCam	Ab56416	1:1000	
p70S6K	Rabbit monoclonal	Cell Signaling	34475	1:1000	
pP70S6K	Rabbit monoclonal	Cell Signaling	9234	1:1000	
β-actin	Mouse monoclonal	Sigma-Aldrich	A2228	1:40000	
RAB5A	Mouse monoclonal	Sigma-Aldrich	R7904		1:100
RAB7	Mouse monoclonal	Sigma-Aldrich	R8779		1:100
LC3B	Rabbit monoclonal	AbCam	Ab192890		1:100
ATG4B	Rabbit polyclonal	AbCam	Ab154843		1:100
ATG5	Rabbit monoclonal	AbCam	Ab108327		1:100
ATG9A	Rabbit polyclonal	AbCam	Ab108338		1:100
ATG16L1	Rabbit polyclonal	AbCam	Ab187671		1:100
Beclin1	Rabbit monoclonal	AbCam	Ab207612		1:100

MEFs and cortical neurons were seeded on coverslips, fixed with a 4% paraformaldehyde (PFA) solution, blocked, and incubated with primary antibodies overnight at 4 °C. The primary antibodies were incubated for 1 h with fluorescent secondary antibodies (1/500) Alexa Fluor 568 anti-mouse (A-10037; Thermo Fisher), Alexa Fluor 488 anti-rabbit (A-11008; Thermo Fisher), and Alexa Fluor anti-rat 568 (A-21247; Thermo Fisher). DAPI (4’,6-diamidino-2-phenylindole) was used for nuclei visualization. Coverslips were mounted in ProLong Gold antifade reagent. Negative controls were performed with IgG antibodies and not presented if there was no contaminating background staining. Images were acquired on a Dragonfly 200 (ANDOR) imaging system using 100x (1.45 numerical aperture) objective. Images were acquired at the same exposure times in the same imaging session using Fusion 2.2 (NEW) software. Quantification was performed after appropriate thresholding using ImageJ software (NIH) in a minimum of 30 cells from at least 3 experiments. Colocalization between the anti-MFSD8 staining (green) and other staining was calculated using ImageJ software. For fluorescence confocal microscopy, cells were fixed in 100% ice-cold methanol. Samples were blocked in 2% BSA in PBS + 0.05% (v/v) Tween 20 (all from Sigma). Antibodies were diluted in blocking buffer and incubated overnight at 4 °C. Secondary anti-bodies (1:500, all Alexa Fluor) were applied for 1 h at room temperature. Immuno-stained cells were mounted with DAPI and analyzed using the Leica Stellaris confocal microscope.

### ApoTracker Flow cytometry

NPCs were harvested and washed with PBS. Cells were incubated with eBioscience™ Fixable Viability Dye eFluor® 780 (65-0865, ThermoFisher) at 1 µL/mL of cells in azide-free and serum/protein-free PBS for 20–30 min at 4 °C. Cells were washed with PBS and centrifuged at 300 × *g* for 5 min. Cell pellet was resuspended in 100 µL of cell staining solution (2% BSA in PBS) and incubated with 400 nM Apotracker™ Green (427402, BioLegend®) (diluted in staining solution) for 20 min at room temperature. Another cell wash with PBS was conducted and cells were fixed with cell fixation buffer (4% PFA) for 15-20 min at room temperature. Cells were washed with PBS and 200–500 µL of PBS was added to the cell pellets for flow cytometry analysis in Falcon® 5 ml Round-Bottom Tubes. A MACSQuant from Miltenyi Biotec was used as a flow cytometer. Apotracker™ Green was excited using the blue 488 nm laser, and the fluorescence emission was collected using a 525/50 nm laser. The eBioscience™ Fixable Viability Dye eFluor® 780 was excited using the red 638 nm laser, and the fluorescence emission was collected using a 785/62 nm laser. The obtained data were analyzed using FlowLogic software.

### Lentiviral production

A CLN7 coding sequence fused to mScarlet3 was synthesized as a double-stranded DNA fragment and cloned into a pCCL lentiviral backbone using standard molecular cloning techniques. HEK 293 T cells were plated at a density of 1 × 106 cells per cm^2^ 24-h before transfection. Lentiviral packaging plasmids pCCL (carrying mScarlet3-CLN7 transgene), pMDLg.RRE, pRSV-Rev, and pMD2G (from Didier Trono - Addgene plasmids 12251, 12253, 12259) were mixed in a 4:2:1:1 ratio and diluted in Opti-MEM™ before combining with an equal volume of Opti-MEM™ containing 1 μM branched PEI. Complexes were incubated for 10 min at room temperature before introducing to plated HEK 293 T cells. The transfection mixture was removed from the cells after 4 h and replaced with 18 mL of DMEM with 10% FBS. Medium was collected 48-h after transfection and filtered with a cellulose acetate membrane (0.22 μm pore) to remove cell debris. The filtered medium was centrifuged at 4500 × g at 4 °C for 18-h to pellet vector material. The pellet from each 40 mL crude harvest was resuspended in 100 μL of PBS, to achieve 400-fold concentration.

### Transcriptomic analysis

#### Cell lysis and RNA Purification

Total RNA was extracted and purified from BD and WT iNPC using the QIAGEN RNeasy mini kit, quantified using a Nanodrop spectrophotometer and for quality and integrity using RNA ScreenTape on the Agilent TapeStation system. To prepare the RNA for sequencing, the Oxford Nanopore Technologies PCR-cDNA Barcoding Kit (SQK-PCB111.24) was used, performing reverse transcription and strand switching, selection for full-length transcripts by PCR, and adapter addition. Quantification was performed with the Qubit dsDNA HS assay, and the Nanodrop spectrophotometer was used for quality assessment. The D5000 DNA ScreenTape on the Agilent TapeStation System was used for fragment analysis.

#### Sequencing

The prepared library was loaded onto a FLO-PRO002 flow cell and sequencing was carried out using the PromethION 2 Solo. After running for 48 h, the total reads were 19.7 M.

#### Data acquisition and analysis

Initial raw data were acquired as a FAST5 file format, which was basecalled using the Guppy software to create a FASTQ file for each sample. The total number of basecalls across all samples was 10.7 Gb. Differential expression analysis was performed using the EPI2ME software with the wf-transcriptomics workflow, aligning the reads to the reference genome Homo_sapiens.GRCh38.dna.primary_assambly, the reference transcriptome Homo_sapiens.GRCh38.cdna.all, and the reference annotation Homo sapiens. GRCh38.111. The parameters for the differential expression analysis were: minimum gene expression 10, minimum feature expression 3, minimum samples with gene expression 3, minimum samples with feature expression 1.

### Proteomic analysis

#### Cell Lysis

75 cm^2^ Flasks were used to culture cells to sub-confluent cultures (around 6 million cells per flask). NPCs were washed with PBS then lysed using 0.5 ml lysis buffer composed of 1 M TEAB (Triethylammonium bicarbonate) and 0.1% (w/v) SDS (Sodium dodecyl sulfate) for 5 min. Lysates were collected using cell scrapers and aliquots were frozen at −80 °C.

#### iTRAQ labelling

The method was performed following the published protocols by Unwin, et al. [63]. Protein concentration was determined by Bradford assay (Bio-RAD). Subsequently, 100 µg of protein from each sample was equalized to 55 µl using 1 M TEAB. 0.1 volumes of 50 mM Dithiothreitol (DTT, Fluka) was added to reduce disulfide bonds and incubated at 60 °C for an hour. Samples were alkylated by 0.05 volumes of 200 mM Iodoacetamide (IAA) at room temperature for 10 min. Proteins were digested using 10 µg trypsin (Sequencing Grade Modified Trypsin, Promega) for 100 µg of protein in each sample. Trypsin was reconstituted in 1 M TEAB so that final SDS concentration in the samples is less than 0.05%, trypsin added to samples and incubated at 37 °C overnight. After digestion samples were dried using a SpeedVac concentrator (Labconco) and frozen at −20 °C.

Dried protein digests were resuspended using 1 M TEAB to 30 µl. iTRAQ reagent vials (AB Sciex) were resuspended with 70 µl isopropanol (Fluka) and transferred to a protein digest. All samples were allowed to react at room temperature for 2 h. Sample volumes were reduced to 30 µl using SpeedVac concentrator to remove isopropanol and stored at −20 °C.

#### High pH reverse phase liquid chromatography fractionation

Fractionation as performed as described in detail in Sharaireh et al. [64]. Peptides were fractionated off-line using an Agilent 1200 series LC system loading 900 ul of sample onto a high pH reversed-phase chromatography column (Agilent ZORBAX 300 Extend-C18 4.6 × 150 mm 3.5 micron). Samples were eluted using a solvent gradient from 5% buffer B and increasing to 30% (buffer B in 35 min, and then to 45% buffer B after 1 min at 45 °C and a flow rate of 700 µl/min. Fractions were collected in a 96-well plate, dried using SpeedVac concentrator and stored at −20 °C.

#### Strong anion exchange desalting and low pH reverse phase liquid chromatography and mass spectrometry (LPLC-MS/MS)

Samples were desalted using POROS 50 HQ strong anion exchange beads (1255911, Thermo Fisher) as previously described in detail in Philips et al. [65]. Collected samples were dried completely using SpeedVac concentrator and samples were stored at −20 °C. Before Mass Spectrometry, the wells in the plate with samples were thawed and each well that contained samples was resuspended in 10 µL of 5% (v/v) acetonitrile/0.1% (v/v) formic Acid. Samples were then transferred to a sample vial and placed on the sample rack for analysis. Low-pH reversed-phase liquid chromatography using a nanoACQUITY UHPLC system (Waters) and BEH C18 Column (1.7 μm, 75 μm × 250 mm) and separated over a 90 min solvent gradient from 3% ACN (v/v) to 40% ACN (v/v) at a flow rate of 300 nl/min and column temperature at 30 °C. Peptides were eluted on-line to TripleTOF® 6600 Quadrupole Time-Of-Flight (QTOF) Mass Analyzer (AB SCIEX). Spectra were collected using a standard IDA method with a 0.1 s MS followed by 10 MS/MS scans over the next one second with iTRAQ collision energies enabled. Each ion was selected a maximum of two times and was dynamically excluded (±50 mmu) for 90 s thereafter.

#### Data analysis

After acquisition of fractioned peptides, raw data files were uploaded to Protein Pilot analysis software to identify and quantify peptides in silico using ProteinPilot version 5.0.1, AB SCIEX. Proteins were searched against a human-specific Uniprot database (Swiss-Prot Human database, containing 20,371 entries, Version number 10_2019). Detection protein threshold was set to as low as possible. Protein and peptide summaries were exported for statistical assessment. Quantified proteins with unused scores equal or above 2 only were included and false discovery rate was 5%. At least one unique peptide of 99% confidence had to be used to identify the protein (Unused score above 2). Log_2_ for the ratio between samples were used to calculate the fold changes. For the analysis of all the four samples simultaneously across the 3 mass-spectrometry runs identified peptides were coalesced to protein-level quantifications and statistical testing for differential expression performed using v1.0.0 of the in-house developed software ‘BayesProt’. An earlier version of this technique was presented in Freeman et al. [66], which combined Protein-Pilot (AB SCIEX) sample normalization (‘bias correction’) with a hierarchical Bayesian modeling to assess the quantification reliability of each feature and peptide across assays so that only those in consensus influence the resulting protein group quantification strongly. Similarly, unexplained variation in each individual assay is captured, providing both a metric for quality control and automatic down weighting of suspect assays. Each protein group-level quantification were outputs from the software and is accompanied by the standard deviation of its posterior uncertainty. This integrated a flexible differential expression analysis subsystem with false discovery rate control based on the popular MCMCglmm package for Bayesian mixed-effects modeling (MCMCglmm R Package [Hadfield, Jarrod D. “MCMC methods for multi-response generalized linear mixed models: the MCMCglmm R package.” Journal of Statistical Software 33.2 (2010): 1e22.]). Method details are described by Xu, J et al. in data processing and Phillips, A et al. [65].

### CLN7 co-IP mass spectrometry

The co-immunoprecipitation of endogenous CLN7/MFSD8 in SH-SY5Y cells was conducted following the protocol published by Lagundžin D et al. [67]. The CLN7 polyclonal antibody raised against L9 (351-421aa) (Proteintech) was used for these experiments. Mass spectrometry sample preparation, data acquisition and analysis were performed as previously described [68]. In brief, samples were prepared for in-gel digestion following the gel-top analysis methodology. 40 µL of the CLN7 immunoprecipitated protein was mixed with a 5× loading buffer and incubated at 95.5 °C for 5–10 min then subjected to 3 min electrophoresis (120 V) on a 10% SDS-PAGE separating gel, stained with InstantBlue™ Coomassie Stain overnight and washed in H_2_O. In-gel tryptic digestion in a perforated 96-well plate was performed, peptides desalted, and mass spectrometry analysis conducted by the University of Manchester Biological Mass Spectrometry Core Facility (RRID: SCR_020987). Protein identifications were validated using statistical algorithms at both the peptide and protein level implemented in Scaffold (version 4.4.7, Proteome Software) and protein abundance was calculated as spectral counts as reported by Scaffold analysis.

Datasets were analysed using the online bioinformatic tools available via the Database for Annotation, Visualization and Integrated Discovery (DAVID; http://david.abcc.ncifcrf.gov/home.jsp). Ontologies with enrichment value ≥ 1.5, Bonferroni-corrected *P* < 0.05, EASE score (modified Fisher Exact *P*-value) <0.05 and at least two genes per term were considered and displayed as a Bubble plot using SRplot (http://www.bioinformatics.com.cn/srplot).

### Statistical analyses

All data are presented as means ± SEM. *n* numbers, statistical tests and *p* values for each experiment are detailed in the respective figure legends. All statistical analysis was performed using GraphPad Prism (GraphPad Software, San Diego, California USA). Unless otherwise stated the following convention is used to denote significance - *p ≤ 0.05, **≤0.01, ***≤0.001 and ****≤0.0001. Details of sample sizes and individual p-values are listed in Table 8.

**Table 8:** Statistical tests, group sizes and *p*-values.

Figure	Group	*N*	Sample Size	Statistical Test	p-value
1 C	CLN7 ICC (CTCF)	6	iNPC^WT^ = 6 iNPC^BD1^ = 6 iNPC^BD2^ = 6	One-way ANOVA	iNPC^WT^ vs iNPC^BD1^ = 0.0436
2B	CLN7 (CTCF)	3	iNPC^WT^ = 3 iNPC^BD1^ = 3 iNPC^BD2^ = 3	One-way ANOVA	iNPC^WT^ vs iNPC^BD1^ = iNPC^WT^ vs iNPC^BD2^ = iNPC^WT^ + Baf vs iNPC^BD1^ + Baf = iNPC^WT^ + Baf vs iNPC^BD2^ + Baf =
2 C	LysoTracker (CTCF)	3	iNPC^WT^ = 3 iNPC^BD1^ = 3 iNPC^BD2^ = 3	One-way ANOVA	iNPC^WT^ vs iNPC^BD1^ = iNPC^WT^ vs iNPC^BD2^ =
3E	qRT-PCR	3	iNPC^WT^ = 3 iNPC^BD1^ = 3 iNPC^BD2^ = 3	Unpaired t-test	iNPC^BD1^ vs iNPC^WT^: CHCHD2, *p* < 0.0001, TOX2, *p* = 0.035, CRYAB, *p* < 0.0001, S100A4, *p* < 0.0001, FABP3, *p* < 0.0001, IFG2, *p* < 0.0001, IGF2R, *p* < 0.0001, IGFBP3, *p* < 0.0001, IGFBP5, *p* = 0.0002, IGFBP6, *p* = 0.093 iNPC^BD2^ vs iNPC^WT^: CHCHD2, *p* < 0.0001, TOX2, p + 0.0002, CRYAB, *p* = 0.0479, S100A4, *p* = 0.0086, FABP3, *p* < 0.0001, IFG2, *p* < 0.0001, IGF2R, *p* = 0.8266, IGFBP3, *p* < 0.0001, IGFBP5, *p* = 0.0176, IGFBP6, *p* < 0.0001
4B	Autophagy ICC (CTCF)	6	iNPC^WT^ = 6 iNPC^WT^ + Baf = 6 iNPC^BD1^ = 6 iNPC^BD1^ + Baf = 6	One-way ANOVA	Listed on graphs
5B	Mito Mem Pot	8	iNPC^WT^ = 6 iNPC^BD1^ = 6	Two-tailed t-test	iNPC^WT^ vs NPC^BD1^ = 0.0003, ***
5 C	MitoSOX	8	iNPC^WT^ = 6 iNPC^WT^ + Baf = 6 iNPC^BD1^ = 6 iNPC^BD1^ + Baf = 6	Two-tailed t-test	iNPC^WT^ vs iNPC^BD1^ < 0.0001, ****
5D	MitoStress Test	6	iNPC^WT^ = 6 iNPC^WT^ + Baf = 6 iNPC^BD1^ = 6 iNPC^BD1^+ Baf = 6	Unpaired t-test	Basal Respiration iNPC^WT^ +Baf vs iNPC^BD1^ +Baf = 0.0007, *** Cln7^WT^ +Baf vs Cln7^KO^ +Baf = 0.013, * Maximal Respiration Cont NPC +Baf vs iNPC^BD1^ +Baf = 0.0066, ** Cln7^WT^ +Baf vs Cln7^KO^ +Baf = 0.0450, * Proton Leak iNPC^WT^ +Baf vs iNPC^BD1^ +Baf = 0.0045, ** Cln7^WT^ +Baf vs Cln7^KO^ +Baf = 0.0043, ** ATP Production iNPC^WT^ +Baf vs iNPC^BD1^ +Baf = 0.0012, ** Cln7^WT^ +Baf vs Cln7^KO^ +Baf = 0.0452, *
5E	NADH/NAD+ Assay	6	iNPC^WT^ = 6 iNPC^WT^ + Baf = 6 CLN7 NPC = 6 CLN7 NPC + Baf = 6	One-tailed t-test	iNPC^BD1^ vs iNPC^BD1^ +Baf = 0.021, * iNPC^WT^ +Baf vs iNPC^BD1^+Baf = 1.467 × 10^−4^, ****
5 F	Apotracker	3	iNPC^WT^ = 3 (3) iNPC^WT^ + Baf = 3 (3) iNPC^BD2^ = 3 (3) iNPC^BD2^+ Baf = 3 (3) iNPC^BD1^ = 3 (3) iNPC^BD1^+ Baf = 3 (3)	One-tailed t-test	iNPC^WT^ vs iNPC^WT^ +Baf *p* = n.s. iNPC^BD2^ vs iNPC^BD2^+Baf *p* = 1.216×10^-5^, **** iNPC^BD1^vs iNPC^BD1^+Baf *p* = 1.684 × 10^−5^, ****

## Supplementary information

Supplementary Figure 1.

Supplementary Figure 2.

Supplementary Figure 3.

Supplementary Figure 4.

Supplementary Figure 5.

Dataset 1

## Data Availability

The proteomics datasets generated during and/or analysed during the current study are available in the PoteomeXchange repository under the accession code: PXD033069. The RNA-seq datasets generated during and/or analysed during the current study are available in the NCBI Sequence Read Archive repository under the accession code: PRJNA1314567.
