# Supplementary figures and images for: CLN7 protein functions at the interface between endolysosomes and stress granules to promote cell survival
Source: Cell Death Dis. 2025 Oct 31;16(1):772. doi: 10.1038/s41419-025-08063-4 (PMC12579238; doi:10.1038/s41419-025-08063-4)

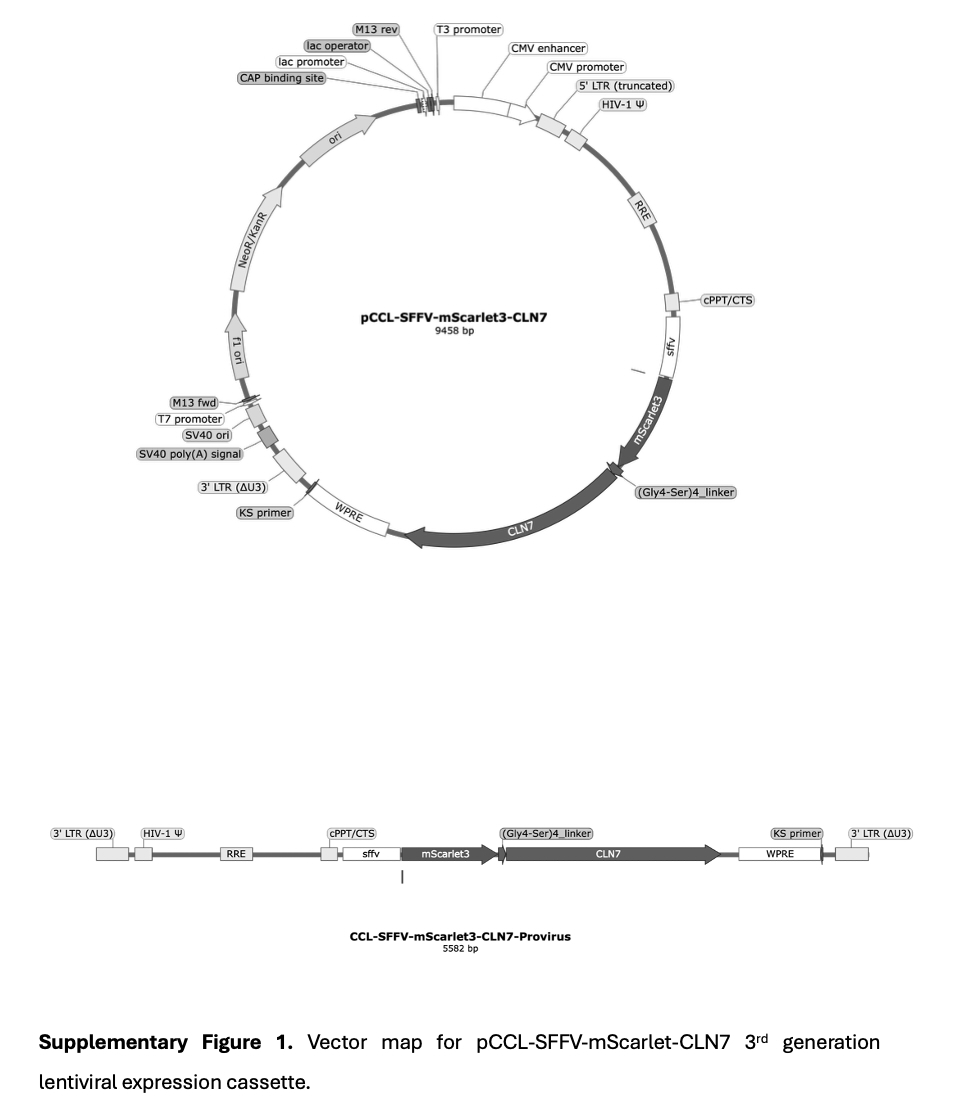

Supplement: Supplementary file 1 — Supplementary Figure 1. [file 41419_2025_8063_MOESM1_ESM.jpg]

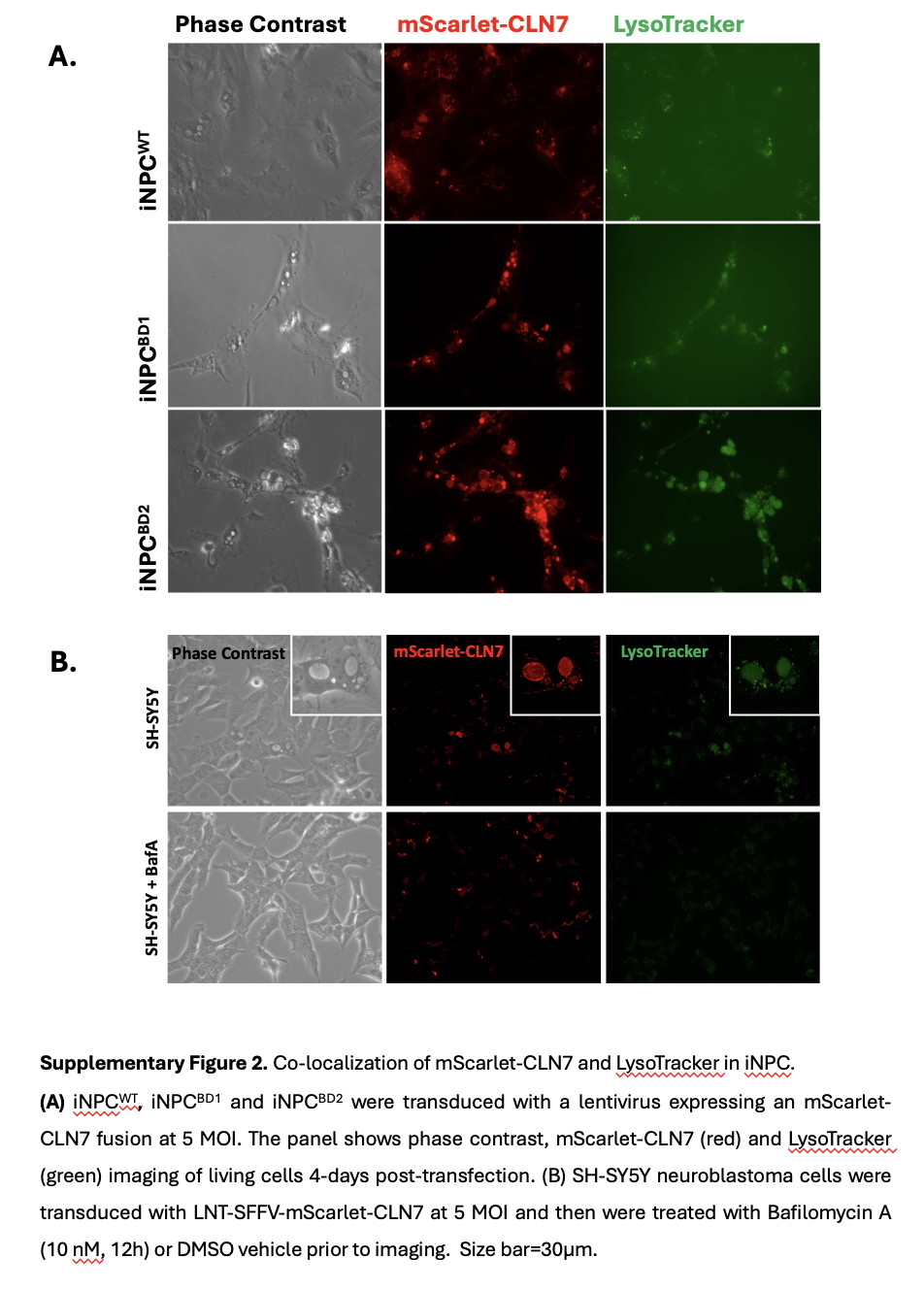

Supplement: Supplementary file 2 — Supplementary Figure 2. [file 41419_2025_8063_MOESM2_ESM.jpg]

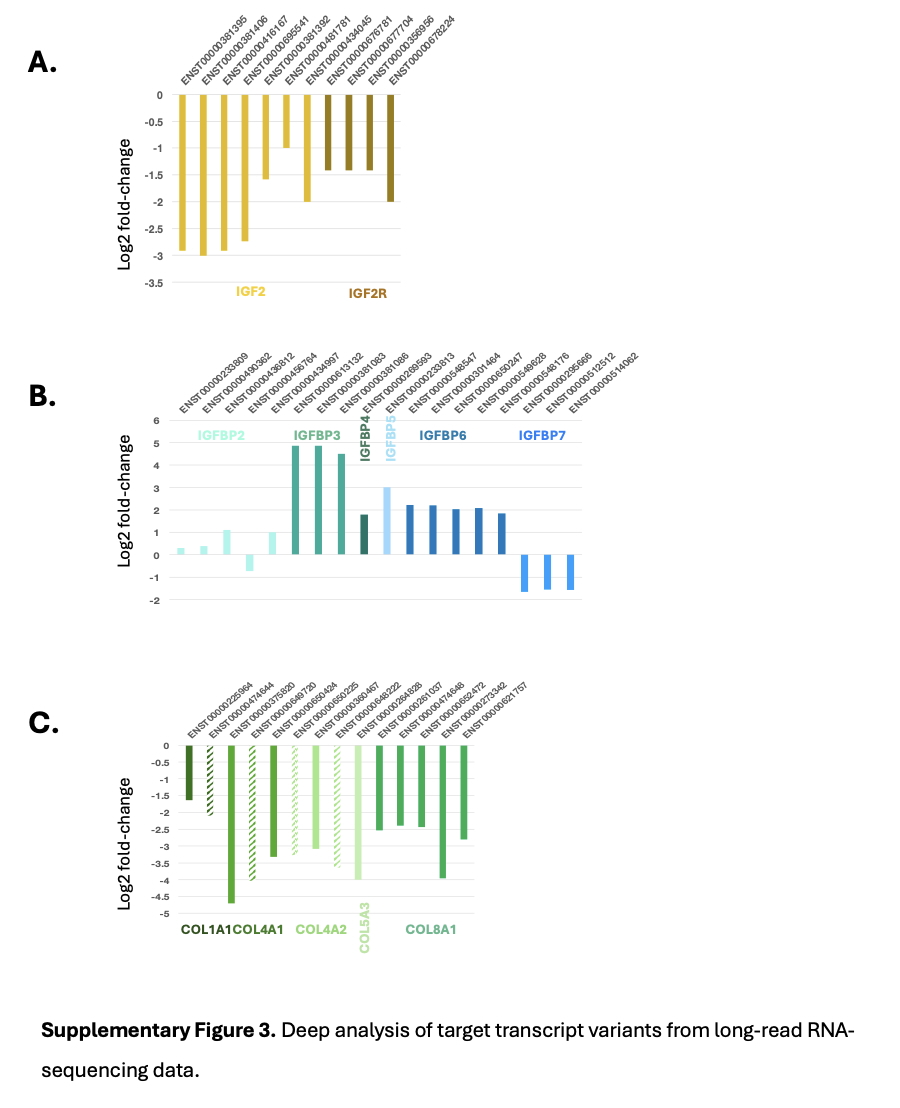

Supplement: Supplementary file 3 — Supplementary Figure 3. [file 41419_2025_8063_MOESM3_ESM.jpg]

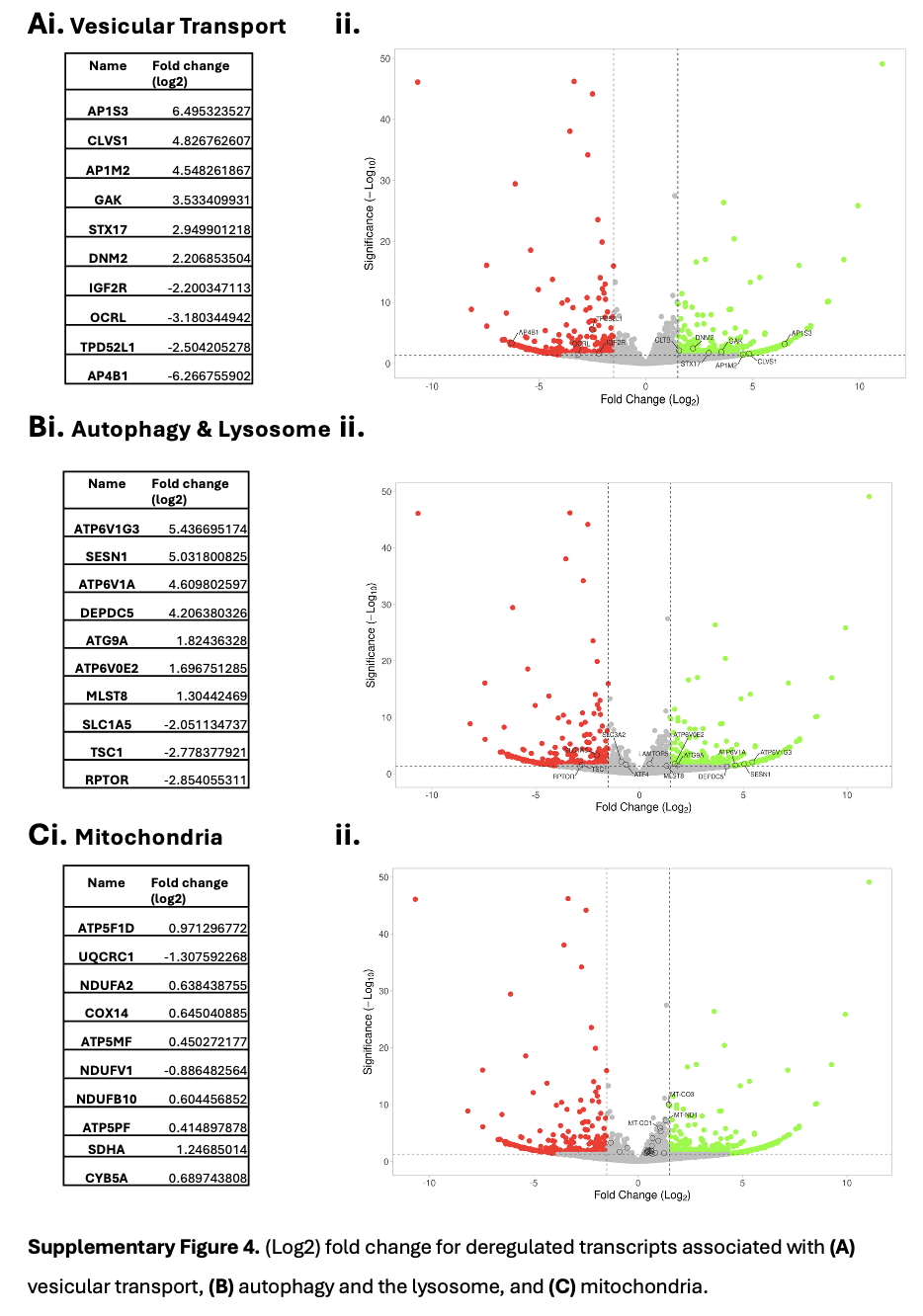

Supplement: Supplementary file 4 — Supplementary Figure 4. [file 41419_2025_8063_MOESM4_ESM.jpg]

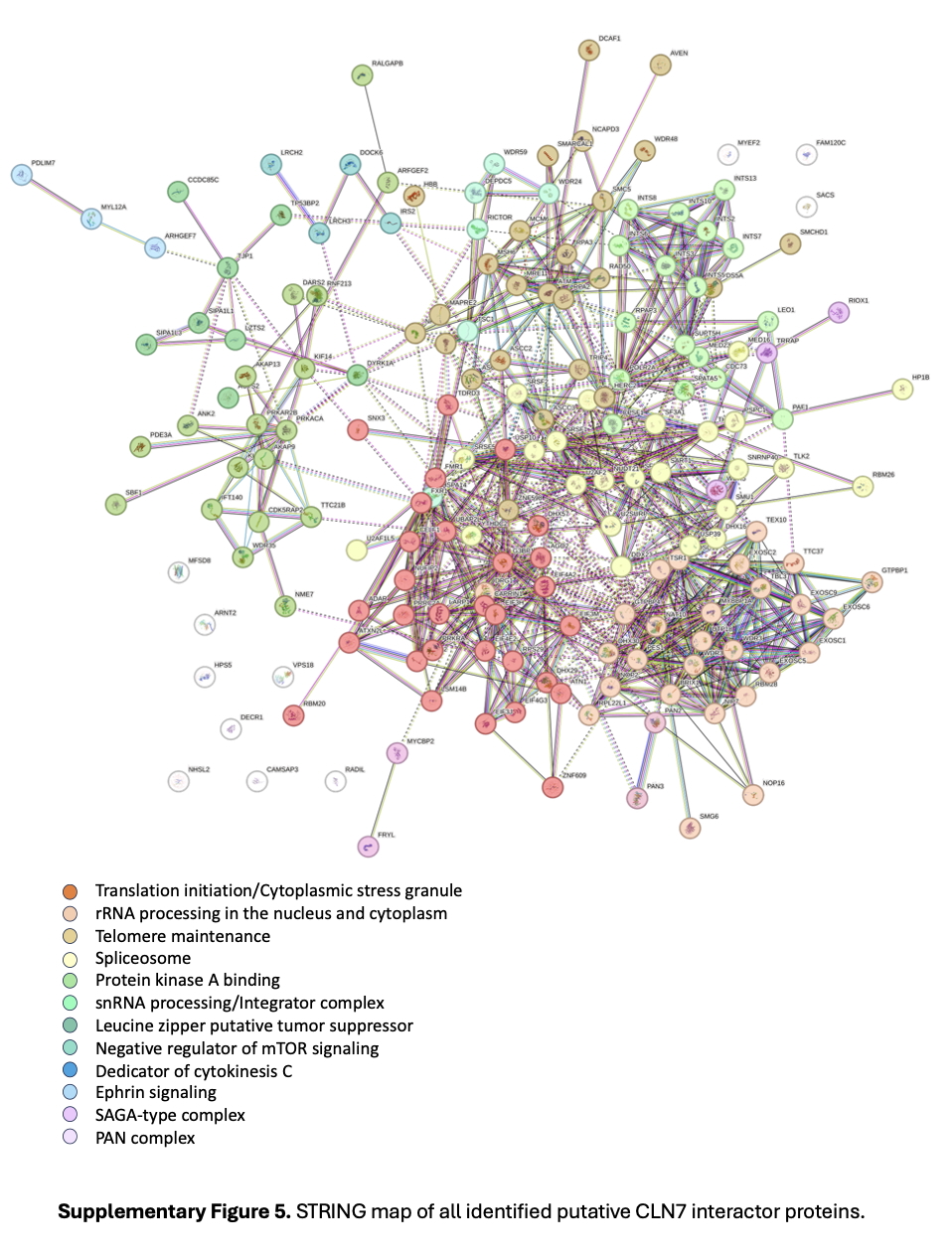

Supplement: Supplementary file 5 — Supplementary Figure 5. [file 41419_2025_8063_MOESM5_ESM.jpg]
